# Phylogenetic analysis and embryonic expression of panarthropod Dmrt genes

**DOI:** 10.1186/s12983-019-0322-0

**Published:** 2019-07-02

**Authors:** Virginia Panara, Graham E. Budd, Ralf Janssen

**Affiliations:** 1 Department of Earth Sciences, Palaeobiology, Uppsala University, Villavägen 16, Uppsala, Sweden; 2 Present address: Department for Immunology, Genetic and Pathology, Rudbeckslaboratoriet, Dag Hammarskjölds väg 20, Uppsala, Sweden

**Keywords:** Doublesex, Sexual differentiation, DMRT, Arthropoda, Panarthropoda, Onychophora, *Tribolium*, *Parasteatoda*, *Glomeris*, *Euperipatoides*, Neo-functionalization

## Abstract

**Background:**

One set of the developmentally important Doublesex and Male-abnormal-3 Related Transcription factors (Dmrt) is subject of intense research, because of their role in sex-determination and sexual differentiation. This likely non-monophyletic group of Dmrt genes is represented by the *Drosophila melanogaster* gene *Doublesex* (*Dsx*), the *Caenorhabditis elegans Male-abnormal-3* (*Mab-3*) gene, and vertebrate Dmrt1 genes. However, other members of the Dmrt family are much less well studied, and in arthropods, including the model organism *Drosophila melanogaster*, data on these genes are virtually absent with respect to their embryonic expression and function.

**Results:**

Here we investigate the complete set of Dmrt genes in members of all main groups of Arthropoda and a member of Onychophora, extending our data to Panarthropoda as a whole. We confirm the presence of at least four families of Dmrt genes (including *Dsx*-like genes) in Panarthropoda and study their expression profiles during embryogenesis. Our work shows that the expression patterns of Dmrt11E, Dmrt93B, and Dmrt99B orthologs are highly conserved among panarthropods. Embryonic expression of *Dsx*-like genes, however, is more derived, likely as a result of neo-functionalization after duplication.

**Conclusions:**

Our data suggest deep homology of most of the panarthropod Dmrt genes with respect to their function that likely dates back to their last common ancestor. The function of *Dsx* and *Dsx*-like genes which are critical for sexual differentiation in animals, however, appears to be much less conserved.

**Electronic supplementary material:**

The online version of this article (10.1186/s12983-019-0322-0) contains supplementary material, which is available to authorized users.

## Background

Dmrt (Doublesex and Male-abnormal-3 Related Transcription factor) genes represent a group of transcription factors that are characterized by the presence of an unusual zinc-finger motif called the DM domain (Doublesex/Male-abnormal-3 domain) ([1] Erdman and Burtis 1993). The first Dmrt gene to be proposed and identified was *Drosophila melanogaster Doublesex* (*Dsx*), a gene that is involved in sex determination in the fly ([2] Hildreth 1965, [3] Burtis and Baker 1989), but Dmrt genes represent an ancestral class of developmental genes that must have evolved before the appearance of bilaterian animals: they are present in cnidarians, placozoans and ctenophores ([4] Miller et al. 2003, [5] Wexler et al. 2014, [6] Chen et al. 2016).

Dmrt genes have been intensively investigated because of their various functions in sex determination and differentiation, and have been identified in various animal groups such as vertebrates (e.g. [7] Matsuda et al. 2007, [8] Yoshimoto et al. 2008, [9] Matson et al. 2010, [10] Su et al. 2015), a cephalochordate ([11] Wang et al. 2012), a tunicate ([11] Wang et al. 2012), some crustaceans ([12] Kato et al. 2008, [13] 2011, [14] Zhang and Qiu 2010, [15] Chandler et al. 2017, [16] Nong et al. 2017, [17] Chebbi et al. 2019), insects (e.g. [18] Oliveira et al. 2009, [19] Kijimoto et al. 2012, [20] Gotoh et al. 2014, [21] 2016, [22] Komata et al. 2016, [23] Price et al. 2015, [24] Xu et al. 2017), a planarian ([25] Chong et al. 2013), and the model nematode *Caenorhabditis elegans* ([26] Shen and Hodgkin 1988, [27] Mason et al. 2008, [28] Siehr et al. 2011). However, despite the great amount of research undertaken on Dmrt genes and their function(s) in sex determination and differentiation, data on arthropods are mostly restricted to insects (and a few crustaceans) and the sex-determining factor *Doublesex* (*Dsx*). In fact, neither expression nor function of the other Dmrt genes has been investigated in detail even in *Drosophila*, and research on panarthropod (i.e. arthropods, tardigrades and onychophorans) Dmrt genes (including *Dsx*) outside Pancrustacea (i.e. crustaceans and insects together) is almost completely lacking. Furthermore, studies on *Dsx* and other Dmrt genes in Pancrustacea mostly focus on their role in adults or sub-adults, and virtually no data exist on their expression patterns or potential functions during embryogenesis, although there are some studies investigating transcript levels in embryos and embryonic tissues, but without providing any detailed data on transcript location (e.g. [29] Morrow et al. 2014).

We have therefore studied the embryonic expression patterns of the full complement of Dmrt genes in three arthropod species representing Insecta (the red flour beetle *Tribolium castaneum*); Myriapoda (the pill millipede *Glomeris marginata*) and Chelicerata (the common house spider *Parasteatoda tepidariorum*); and the onychophoran *Euperipatoides kanangrensis*. Our data thus represent the first comprehensive study of embryonic Dmrt gene expression patterns in Panarthropoda as a whole. Our phylogenetic analysis clearly groups panarthropod Dmrt genes into three families, Dmrt11E/Dmrt2/terra, Dmrt93B/Dmrt3 and Dmrt99B/Dmrt4,5 (Table 1), and identifies possible Dsx orthologs in at least the spider. We find that most of the identified Dmrt genes are often expressed in a tissue- or structure-specific pattern. For orthologs of Dmrt11E, Dmrt93B and Dmrt99B, these patterns are highly conserved in all panarthropod species including *Drosophila* suggesting ancestral function(s) in panarthropod development that likely dates back to their last common ancestor. In contrast, *Doublesex-like* genes are either not expressed during embryogenesis, or show lineage-specific expression patterns, likely due to neo-functionalization after duplication. A hallmark of insect Dsx genes is their alternative splicing. We detected splice variants of several Dmrt genes, including Dsx genes, and found that at least some of these are expressed in different embryonic structures

**Table 1:** Alternative names of DMRT genes as used in the fly *Drosophila melanogaster*, the nematode worm *Caenorhabditis elegans*, and in verterbrates

*Drosophila* (Panarthropoda)	*Caenorhabditis*	Vertebrata
*doublesex (dsx)*	---	---
---	*mab-3*	
---	---	*Dmrt1*
*Dmrt11E*	---	*Dmrt2/terra*
*Dmrt93B*	*dmd-4*	*Dmrt3*
*Dmrt99B*	*dmd-5*	*Dmrt4* and *Dmrt5*

Based on phylogenies in Volff et al. (2003) and Wexler et al. (2014)

.

## Methods

### Animal husbandry and fixation of embryos

Embryos were obtained and fixed for in-situ hybridization experiments as described in [30] Grossmann and Prpic (2012) (for the red flour beetle *Tribolium castaneum*), [31] Janssen et al. (2004) (for the common pill millipede *Glomeris marginata*), [32] Prpic et al. (2008) (for the common house spider *Parasteatoda tepidariorum*), and [33] Hogvall et al. (2014) (for the velvet worm *Euperipatoides kanangrensis*).

Developmental stages were determined after the staging-systems provided in [34] Strobl and Stelzer (2014) (*Tribolium*), [31] Janssen et al. (2004) (*Glomeris*), [35] Mittmann and Wolff (2012) (*Parasteatoda*), and [36] Janssen and Budd (2013) (*Euperipatoides*).

### RNA extraction, gene cloning, whole mount in-situ hybridization, and nuclear counter staining

For all investigated species, total RNA from a mix of embryos of different developmental stages was extracted using TRIZOL (Invitrogen), and reverse transcribed into cDNA. Fragments of candidate genes were amplified by means of RT-PCR. Gene-specific primers were designed based on published sequence information and sequenced embryonic transcriptomes of *Glomeris* ([37] Janssen and Posnien 2014) and *Euperipatoides* ([36] Janssen and Budd 2013). Nested PCRs were run with internal primers, using a first PCR as template. Primer sequences are summarized in Additional file 1: Table S1. All investigated gene fragments were cloned into the PCRII vector (Invitrogen) and sequenced on an ABI3730XL automatic sequencer (Macrogen, Seoul, South Korea). Gene identification-numbers are listed in Additional file 2: Table S2. Colorimetric in-situ hybridizations for all investigated species were performed as described in [38] Janssen et al. (2018). For confocal microscopy, embryos were stained with SIGMAFAST Fast Red TR/NaphtolAS-MX (SIGMA) instead of BM Purple (ROCHE). Cell nuclei were visualized by either incubation of the embryos in 3–5 μg/ml of 4–6-diamidino-2-phenylindole (DAPI) or SYBR Green in phosphate buffered saline with 0.1% Tween-20 (PBST-0.1%).

### Phylogenetic analysis

**Fig. 1:**
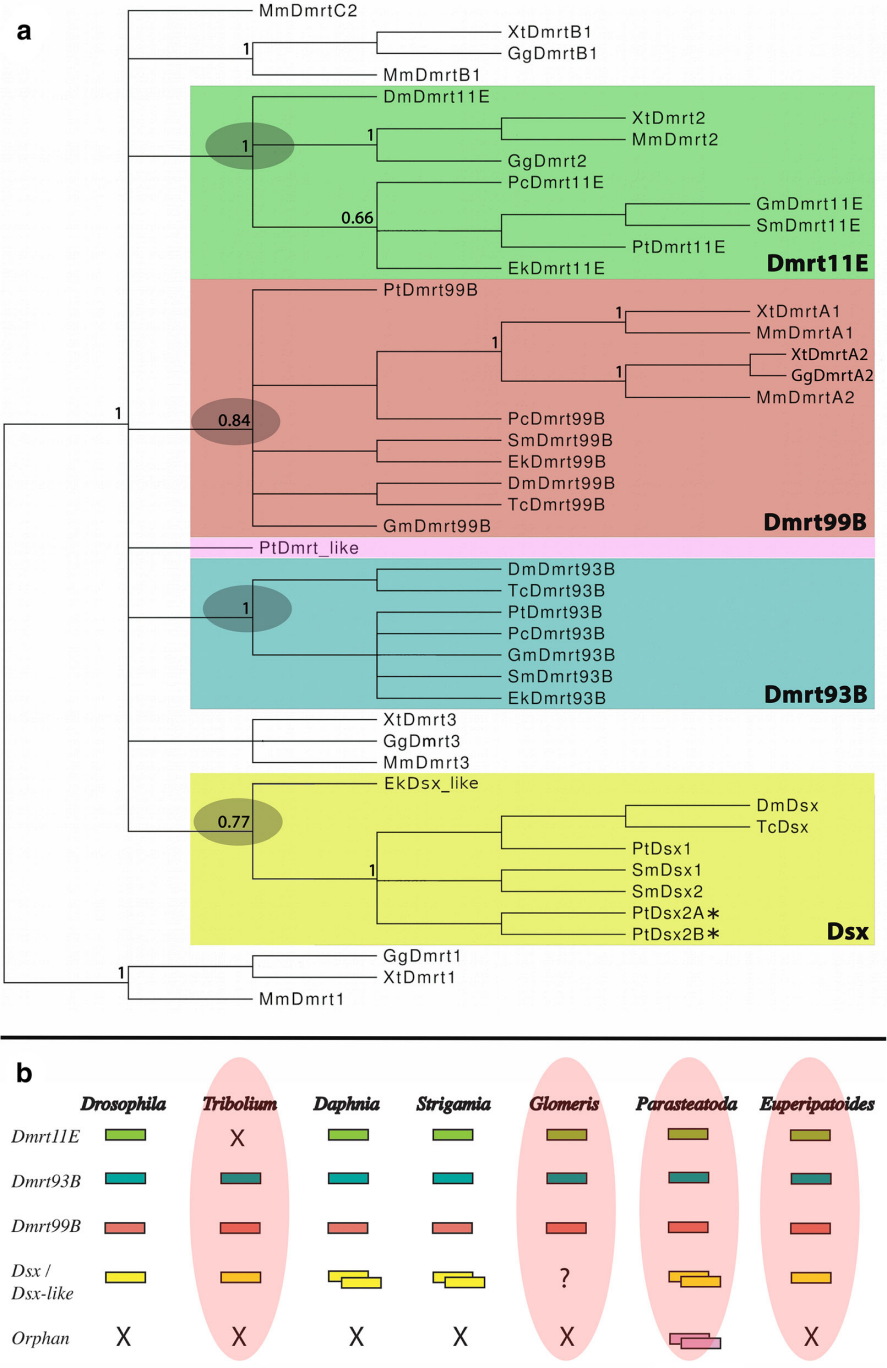
Phylogenetic analysis and gene content. **a** Phylogenetic analysis of Dmrt genes. Species abbreviations: Ek, *Euperipatoides kanangrensis* (Onychophora); Dm, *Drosophila melanogaster* (Hexapoda: Diptera); Gg, *Gallus gallus* (Vertebrata); Gm, *Glomeris marginata* (Myriapoda: Diplopoda); Pc, *Priapulus caudatus* (Priapulida); Pt, *Parasteatoda tepidariorum* (Chelicerata: Araneae); Mm, *Mus musculus* (Vertebrata); Sm, *Strigamia maritima* (Myriapoda: Chilopoda); Tc, *Tribolium castaneum* (Hexapoda: Coleoptera); Xt, *Xenopus tropicalis* (Vertebrata). Green shade: Dmrt11E group. Red shade: Dmrt99B group. Blue shade: Dmrt93B group. Yellow shade: Doublesex (Dsx) group. Magenta shade: Orphan Dmrt gene. Grey shades mark relevant support values for the four monophyletic groups of panarthropod Dmrt genes. Node support is given as posterior probabilities. See text for further information. **b** Content of Dmrt genes in the model arthropod *Drosophila melanogaster*, the here investigated species (red shades), the water flea *Daphnia magna* and the centipede *Strigamia maritima*. Question marks indicate unclear presence of genes (embryonic transcriptome data). ‘X’ indicates missing genes. Each box indicates the presence of one paralog

Reciprocal BLAST searches against sequenced embryonic transcriptomes of *Glomeris* (SRA accession: PRJNA525752) and *Euperipatoides* (SRA accession: PRJNA525753) (applying tblastn), against published protein sequences from *Tribolium* and *Parasteatoda* (applying both blastp and blastx), and the sequenced transcriptome of the priapulid worm *Priapulus caudatus* (SRX507009) were run with the *Drosophila melanogaster* sequences of Dsx, Dmrt11E, Dmrt93b and Dmrt99B, and with a Dmrt gene from the Chinese mitten crab *Eriocheir sinesis* ([14] Zhang and Qiu 2010) to identify Dmrt and Dmrt-like genes. Amino acid sequences of the complete coding regions (Fig. 1, Additional file 6: Figure S3 and Additional file 8: Figure S5) or the Dmrt-domains (DM domains) (Additional file 4: Figure S1, Additional file 5: Figure S2 and Additional file 7: Figure S4) were aligned using T-Coffee followed by manual editing in SeaView ([39] Notredame et al. 2000, [40] Gouy et al. 2010) with default parameters in MacVector v12.6.0 (MacVector, Inc., Cary, NC), or Aliview 1.18.1 for Linux ([41] Larsson, 2014). Phylogenetic analyses were conducted using MrBayes ([42] Huelsenbeck and Ronquist 2001) and a fixed WAG amino acid substitution model with gamma-distributed rate variation across sites (with four rate categories), unconstrained exponential prior probability distribution on branch lengths, and exponential prior for the gamma shape parameters for among-site rate variation was applied. Gene topology was computed applying 2 million cycles for the Metropolis-Coupled Markov Chain Monte Carlo (MCMCMC) analysis (four chains; chain-heating temperature of 0.2). Markov chains were sampled every 200 cycles and default settings of 25% of samples were applied as burn-in. Clade support was calculated with posterior probabilities in MrBayes. Sequence identifiers for published sequences used in the phylogenetic analysis are summarized in Additional file 2: Table S2.

### Data documentation

Bright-field microscopy and documentation of DAPI counterstained embryos were performed using a Leica DC490 digital camera equipped with a UV light source mounted onto a MZ-FLIII Leica dissection microscope. For confocal microscopy, an inverted Leica TCS SP5 confocal microscope was used. For the detection of Fast Red and DAPI signal, the emission wavelengths were 561 nm and 404 nm respectively, and the collected wavelengths were between 600 nm and 642 nm for Fast Red, and between 430 nm and 550 nm for DAPI.

When appropriate, contrast and brightness were adjusted using the image-processing software Adobe Photoshop CS6 for Apple Macintosh (Adobe Systems Inc.) and GIMP 2.10.0 for Linux ([43] Kimball et al. 2018).

## Results

### Sequence analysis

**Fig. 2:**
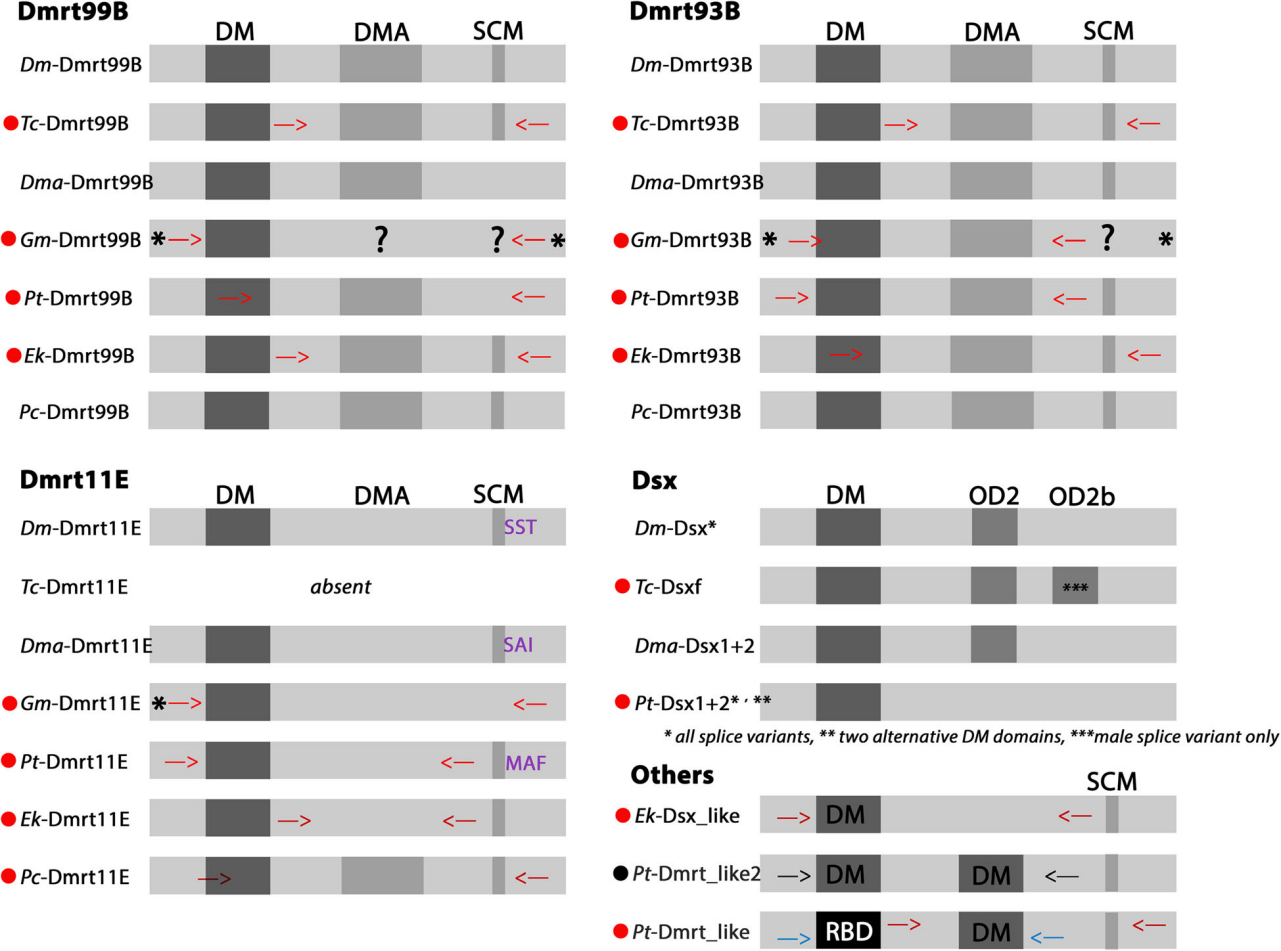
Conserved domains of Dmrt genes. The conserved domains are highlighted: the DM domain, the DMA domain, the oligomerization domain (OD) of pancrustacean Dsx genes, the Short-Conserved-Motif (SMC), and the peculiar reticulocyte binding domain-like motif (RBD). Asterisks (*) mark unknown sequence information. Question marks (?) indicate missing information about presence or absence of a domain. Whenever the core motif (SAF) of the SCM is derived, this is indicated with the different sequence in purple letters next to the SCM-domain box. Position of primers of cloned and sequenced gene fragments is indicated by red arrows. The region marked by the red arrows was used to synthesize anti-sense mRNA probes for in-situ hybridization experiments. Blue arrows mark the region of *Pt-Dmrt_like* that we cloned to verify the presence of the RBD-like motif. Red filled circles in front of gene names indicate genes that have been cloned in this study. The black filled circle indicates that we attempted to amplify this gene by means of RT-PCR (position of primers marked by black arrows), but did not succeed. Locations of primers for the different splice variants of *Tribolium Dsx*, *Parasteatoda Dsx2* and *Glomeris Dmrt11E* are indicated in Fig. 3. Species abbreviations: Dm, *Drosophila melanogaster*; Dma, *Daphnia magna*; Ek, *Euperipatoides kanangrensis*; Gm, *Glomeris marginata*; Pc, *Priapulus caudatus*; Pt, *Parasteatoda tepidariorum*; Tc, *Tribolium castaneum*

We identified three Dmrt genes in *Tribolium* (cf. [5] Wexler et al. 2014), three in *Glomeris*, six in *Parasteatoda*, and four in *Euperipatoides* (Fig. 1a/b). A phylogenetic analysis based on the sequence of the DM domain did not resolve very well, especially with respect to Dmrt99B orthologs and *Euperipatoides Dsx_like* (Additional file 4: Figure S1 and Additional file 5: Figure S2). A likely reason for this is the very limited sequence information and phylogenetic power the DM domain alone provides. The phylogenetic analysis based on the complete open reading frames, including the conserved DMA domain and the “short conserved motif” ([4] Miller et al. 2003, [44] Ottolenghi et al. 2002), however, confidently places all Dmrt genes, except the orphan Dmrt gene *Parasteatoda Dmrt_like*, into four categories of arthropod Dmrt genes (cf. phylogenies provided in [5] Wexler et al. (2014), [45] Pomerantz et al. (2015), [46] Jia et al. 2018) (Fig. 1a, Additional file 6: Figure S3) (note that *Pt-Dmrt_like2* was not used in this phylogenetic analysis because the sequence did not align properly due to the presence of two DM domains). Each of the species investigated here possesses one Dmrt93B gene, one Dmrt99B gene, and one Dmrt11E gene (except for *Tribolium*). We identified one *Tribolium Doublesex* (*Dsx*) gene (cf. [47] Shukla and Palli 2012), but two *Parasteatoda* Dsx genes (*Dsx1* and *Dsx2*). In *Euperipatoides*, we identified one Dmrt gene that clusters with arthropod Dsx genes; but support for this relationship is relatively low and its structure is significantly different from that of arthropod Dsx genes (Figs. 1a and 2). However, because of its position in the phylogenetic tree we named this gene *Euperipatoides Dsx_like*. We could not detect a *Glomeris Dsx* gene, but this may represent an artefact of incomplete transcriptome data, or it could be that *Glomeris Dsx* is expressed at later developmental stages that are not covered by the sequenced transcriptome.

**Fig. 3:**
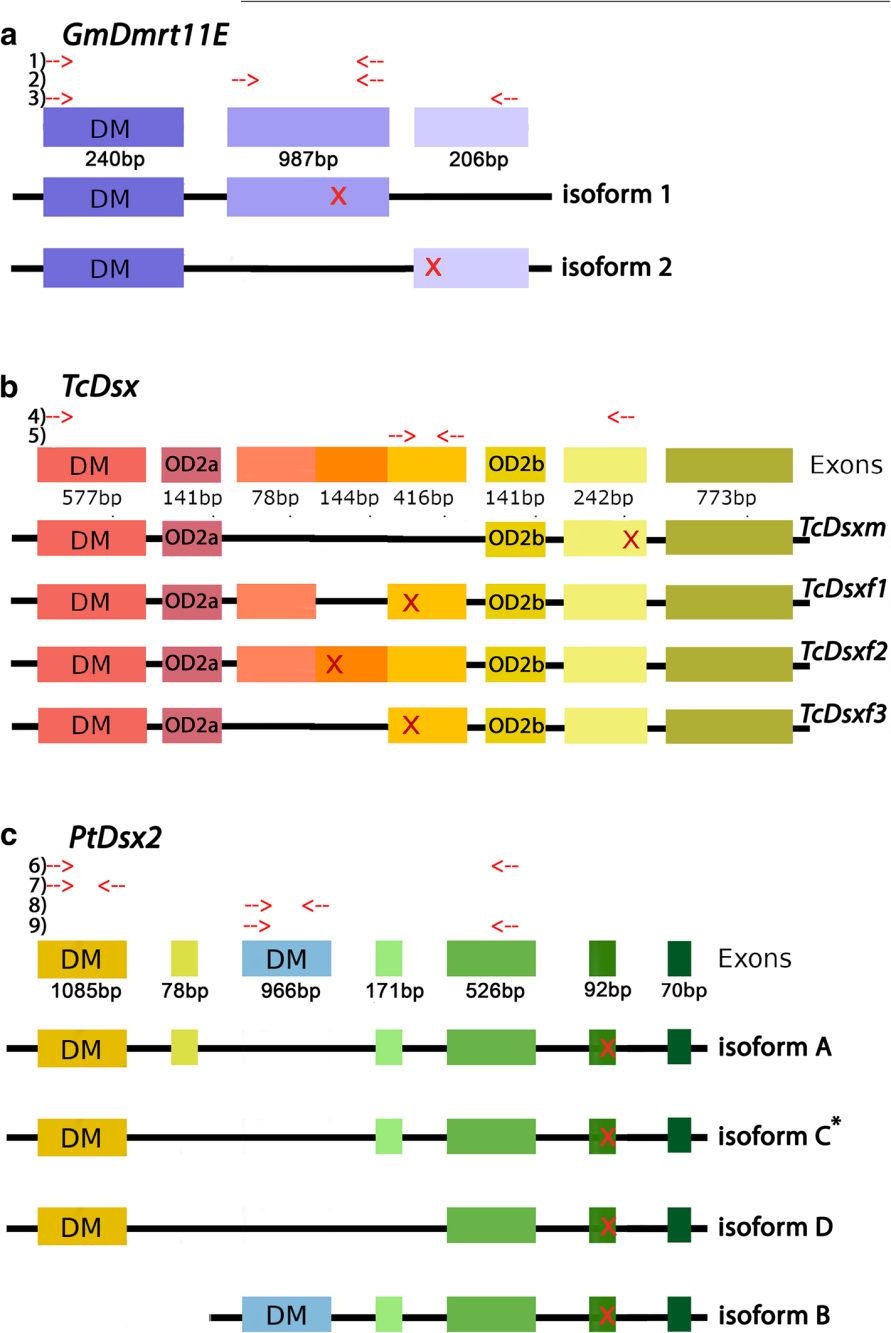
Splice variants of here investigated Dmrt genes. Each coloured box represents one exon. For *GmDmrt11E*, the position of exon 2 and 3 is arbitrary, since genomic data are not available. The position of stop codons is marked by a red ‘X’. The DM domains and the oligomerization domain 2 (OD2a and OD2b) are indicated. Length of exons is given in base pairs. Relative position of primers used to verify splice variants and for subsequent probe synthesis are indicated: 1) long isoform of *Gm-Dmrt11E*; 2) probe for *Gm-Dmrt11E*; 3) short isoform of *Gm-Dmrt11E*; 4) universal probe of *Tc-Dsx*; 5) female-specific probe of *Tc-Dsx*; 6) primers used to confirm presence of isoforms A and D (*note that we could not isolate isoform C (as predicted from genomic data) from our cDNA samples); 7) probe for isoforms A/C/D; 8) probe for isoform B; 9) primers used to confirm presence of isoform B. All PCR fragments were cloned and sequenced

*Glomeris Dmrt11E* is expressed in two isoforms that we confirmed by RT-PCR, cloning and sequencing (Fig. 3a). *Tribolium Dsx* is expressed in four isoforms, one male-specific and three female-specific isoforms ([47] Shukla and Palli 2012). The position of the stop codon of the three female isoforms differs as they are located on different exons (Fig. 3b). *Parasteatoda Dsx2* is expressed in four isoforms, of which three (isoforms A, C and D) contain the same DM domain, but one, isoform B, contains an alternative DM domain (Fig. 3c). Prediction of these isoforms was based on published genome and transcriptome data. We confirmed all isoforms except for isoform C by RT-PCR, cloning and sequencing. Possibly, isoform C is not represented by the embryonic cDNAs we used for RT-PCR, or is expressed at very low levels.

### Gene structure

Typically, Dmrt genes possess one DM domain which encodes a zinc-finger DNA-binding motif ([1] Erdman and Burtis 1993). Some classes of Dmrt genes also contain a so-called DMA domain ([44] Ottolenghi et al. 2002). The function of this domain is not fully understood, but it has been suggested that it may play a role during neurogenesis ([48] Huang et al. 2005, [49] Parlier et al. 2013). Dmrt genes can thus be separated into those with only a DM domain and those with a DM and a DMA domain ([44] Ottolenghi et al. 2002, [11] Wang et al. 2012). However, some Dmrt genes are expressed as isoforms that lack the DM domain, such as an isoform found for *Daphnia* Dmrt99B ([12] Kato et al. 2008). Another conserved domain is the “short conserved motif” (SCM) of unknown function downstream of the DMA domain ([4] Miller et al. 2003). This seven-amino acid long sequence is characterized by three highly conserved amino acids in position two to four (i.e. SAF) ([4] Miller et al. 2003, [12] Kato et al. 2008). For the nematode worm, *Caenorhabditis elegans*, and some species of ants, Dmrt genes with two DM domains have been reported ([50] Volff et al. 2003, [11] Wang et al. 2012, [46] Jia et al. 2018). In principle, all Dmrt genes possess one N-terminal DM domain. Dmrt99B-type and Dmrt93B-type genes also possess a DMA domain downstream of the DM domain while this domain appears to be missing from Dmrt11E-type genes (e.g. [12] Kato et al. 2008). All three Dmrt99B, Dmrt93B and Dmrt11E possess the SCM, although this motif is absent (or highly diverged) in members of at least Dmrt11E genes (e.g. [12] Kato et al. 2008). Doublesex-type Dmrt genes are characterized by the presence of a N-terminal DM domain and a downstream located oligomerization domain (OD2) ([51] An et al. 1996, [52] Bayrer et al. 2005), but they appear to lack a SCM (e.g. [53] Toyota et al. 2013).

We have analysed the gene structure of Dmrt genes identified in our study as well as Dmrt genes from the priapulid worm *Priapulus caudatus* (as a distantly related and slowly evolving ecdysozoan outgroup species (e.g. [54] Webster et al. 2006, [55] Dunn et al. 2008)). For all investigated genes, we found at least one DM domain (Fig. 2). The *Parasteatoda* Dsx2 gene contains two DM domains each of which appear in different isoforms. One of the *Parasteatoda* orphans (*Pt-Dmrt_like2*), however, possesses two DM domains that appear in the same transcript and the other orphan, *Pt-Dmrt_like*, possesses a C-terminal DM domain and an unusual second conserved motif that is similar to the reticulocyte binding domain (RBD) that has only been reported from the parasite *Plasmodium* (reviewed in e.g. [56] Gunalan et al. 2013). The DMA domain is present in all Dmrt99B and Dmrt93B genes. However, we also found a DMA domain in *Priapulus* Dmrt11E suggesting that the ancestral Dmrt11E gene may have possessed a DMA domain that was later lost in the lineage leading to Panarthropoda (Fig. 2). The SCM is present in all Dmrt93B genes as well as most of the Dmrt99B genes, for each gene group with its conserved core sequence (SAF) (e.g. [4] Miller et al. 2003, [12] Kato et al. 2008, [11] Wang et al. 2012) (Fig. 2). In Dmrt11E genes, however, the SCM is divergent in arthropods, but in the onychophoran and the priapulid, this domain is conserved, with its core sequence (SAF) suggesting that the domain has diverged in the lineage leading to Arthropoda (Fig. 2). Insect and at least cladoceran crustacean Dsx genes possess a clearly recognizable oligomerization domain (OD2) ([52] Bayrer et al. 2005, [13] Kato et al. 2011, [53] Toyota et al. 2013), but this domain is not recognized by the algorithms used on BLAST search ([57] Marchler-Bauer et al. 2017) in *Daphnia* Dsx genes and the spider Dsx genes, and we could not identify a possible OD2 domain in *Parasteatoda* (Fig. 2).

### Embryonic expression patterns of panarthropod Dmrt genes

#### Dmrt11E

The genome of *Tribolium* does not contain a *Dmrt11E* gene (see also [5] Wexler et al. 2014).

**Fig. 4:**
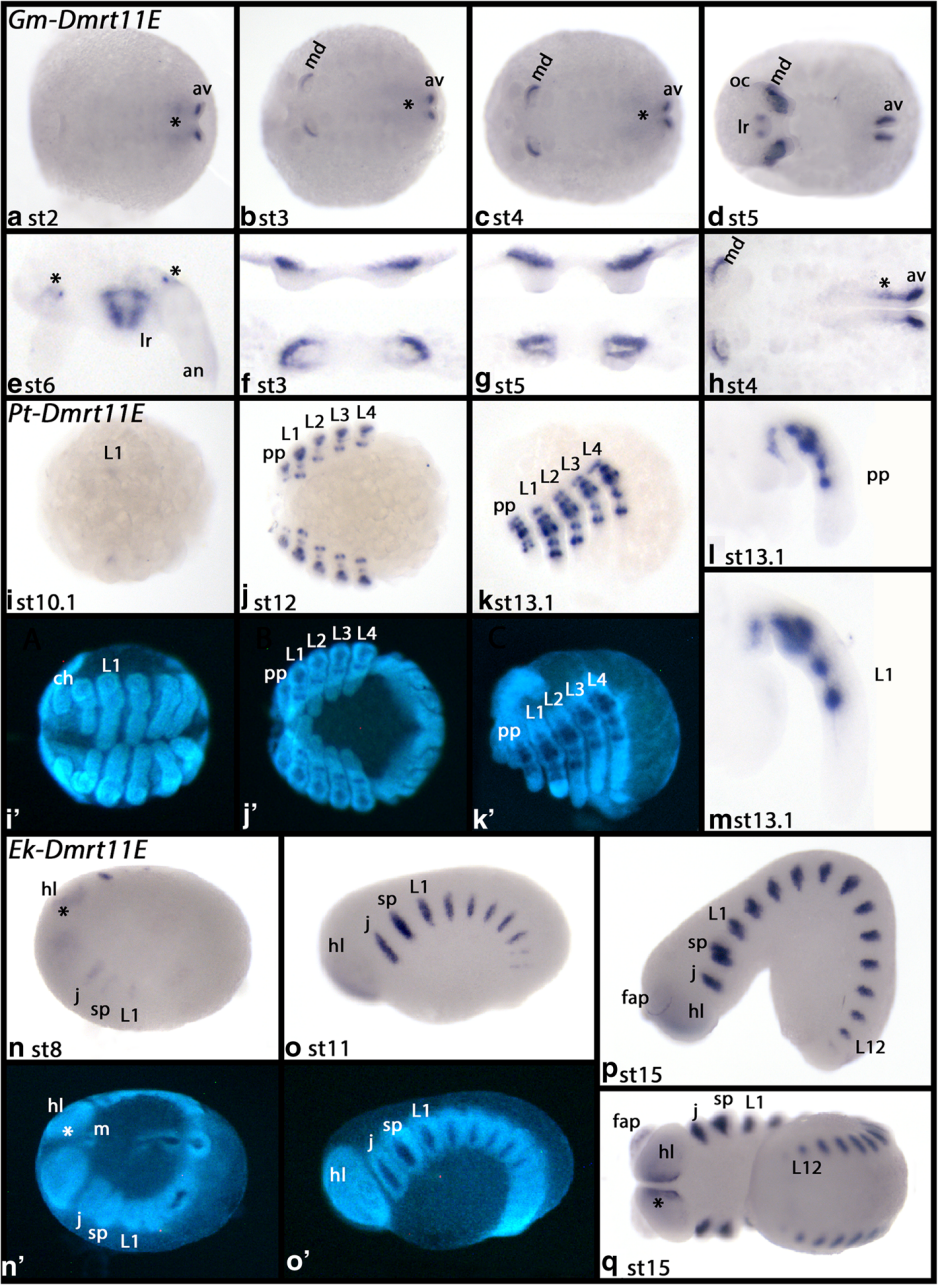
Expression of *Dmrt11E* in *Glomeris ***A-H**, *Parasteatoda ***I-M** and *Euperipatoides ***N-Q**. In all panels, anterior is to the left and ventral views (except for panels E (anterior view), K-M, O, P (lateral views, dorsal up). Developmental stages are indicated. Panels I´-K´ and N´, O´ represent DAPI stained embryos as seen in panels I-K and N, O. Asterisks (*) in panels A-C and H mark expression in the outer lining of the hindgut. Asterisk (*) in panel E mark expression at the base of the antennae. Asterisks (*) in panels N, N´ and Q mark expression anterior to the mouth. Abbreviations: an, antenna; av., anal valve; ch, chelicera; fap, frontal appendage; j, jaw; hl, head lobe; Lx, walking-leg bearing segment number X; lr, labrum; m, mouth; md, mandible; oc, ocular region; pp, pedipalp; sp, slime papilla

In *Glomeris*, *Dmrt11E* is expressed in two isoforms. A probe targeting the specific sequence of the longer transcript was used (Fig. 3a). At stage 2, isoform_1 of *Dmrt11E* is expressed in the anterior mesoderm of the mandibles (Fig. 4B-D, F-H), the mesoderm of the anal valves (Fig. 4A-D, H), and in the outer lining of the developing hindgut (Fig. 4A-C, H). At stage 5, expression appears in the mesoderm of the labrum (Fig. 4D/E). Unfortunately, it was not possible to design a specific probe for the shorter isoform, as the specific sequence is too short to use in in-situ hybridization experiments.

*Parasteatoda Dmrt11E* is first expressed at stage 10.1 in the form of a dot in each of the most anterior walking legs (Fig. 4I). Later, a dotted pattern along the proximal-distal axis appears in the mesoderm of all limbs except for the chelicerae (Fig. 4J-M).

In *Euperipatoides*, *Dmrt11E* is first expressed at stage 8 in the form of a weak expression in the jaw, the slime papilla and the first pair of legs (Fig. 4N). As development progresses, more stripes of expression appear successively in differentiating posterior segments (Fig. 4O/P). This expression is in the mesoderm of the limbs, as confocal microscopy reveals (Additional file 9: Figure S6). At later developmental stages, *Dmrt11E* is also expressed in the ventral lining of the head lobes anterior to the position of the mouth (Fig. 4Q).

#### Dmrt93B

**Fig. 5:**
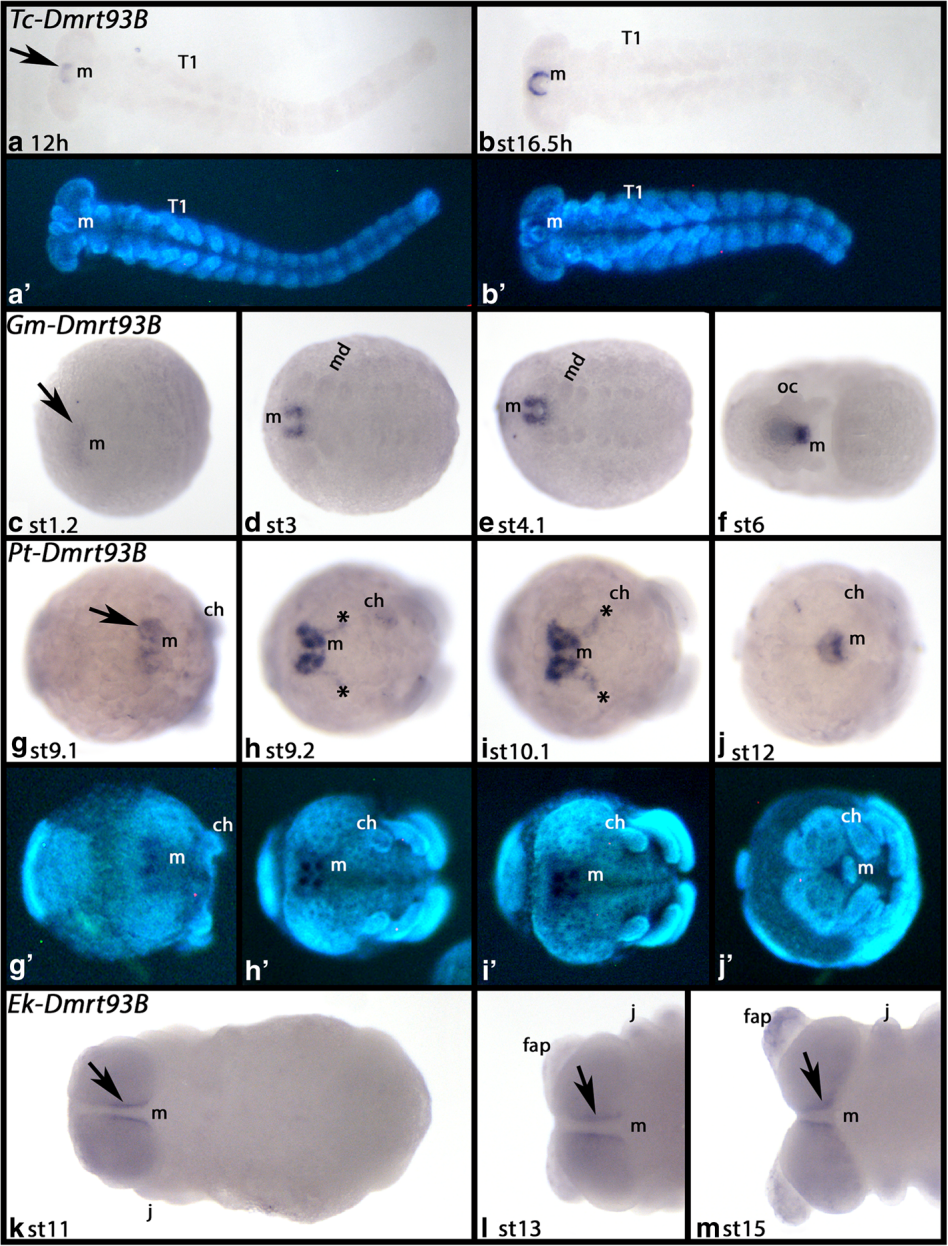
Expression of *Dmrt93B* in *Tribolium* (**A**, **B**), *Glomeris* (**C**-**F**), *Parasteatoda* (**G-J**) and *Euperipatoides* (**K-M**). Developmental stages are indicated. Panels A´, B´ and G´-J´ represent DAPI stained embryos as seen in panels A, B and G-J. In all panels, anterior is to the left. Ventral views. Arrows point to expression anterior to or around the position of the mouth. Asterisks (*) in panels H and I mark protrusions of the expression. Abbreviations as in Fig. 4; T, trunk segment

In all species, *Tribolium*, *Glomeris*, *Parasteatoda* and *Euperipatoides*, *Dmrt93B* is exclusively expressed in tissue in and around the developing mouth (Fig. 5).

#### Dmrt99B

**Fig. 6:**
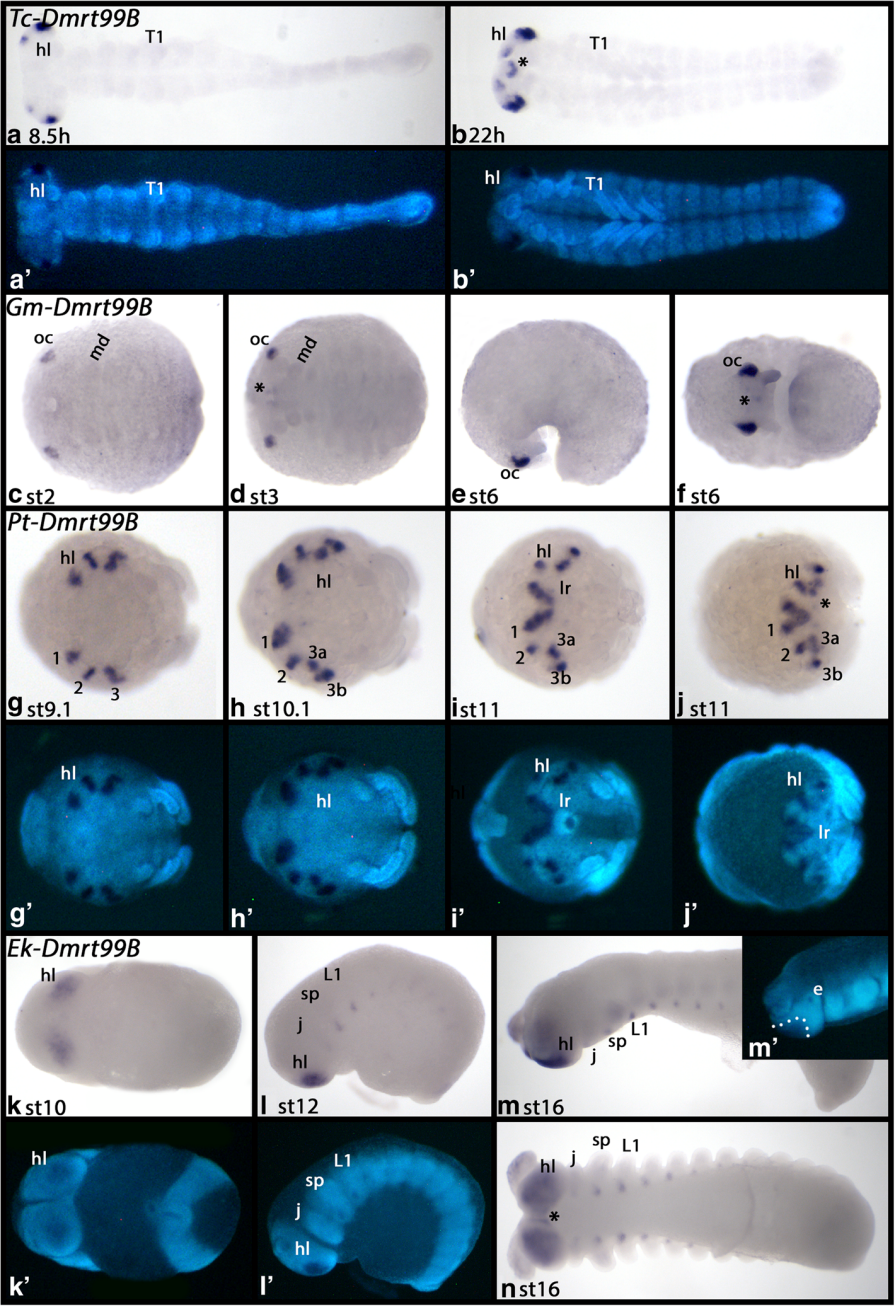
Expression of *Dmrt99B* in *Tribolium* (**A, B**), *Glomeris* (**C-F**), *Parasteatoda* (**G-J**) and *Euperipatoides* (**K-N**). Developmental stages are indicated. Panels A´, B´ and G´-M´ represent DAPI stained embryos as seen in panels A, B and G-M. In all panels, anterior is to the left and ventral views, except panels E, L and M (lateral views, dorsal up), and panels G-J (anterior views). Asterisks (*) mark expression around or in the mouth. Dashed line in panel M´ marks position of expression in the head lobes. Note that the embryo in M´ is the same as shown in panel M, but in a slightly different orientation. Abbreviations as in Fig. 4; roman numerals indicate different domains of spider *Dmrt99B* expression; e, eye

In all species, *Dmrt99B* is predominantly expressed in the developing brain (Fig. 6). In *Tribolium* this is first in the form of four domains in the head lobes (two in each hemisphere), that later transform into six domains (or two domains appear de novo) (Fig. 6A/B). In *Glomeris*, *Dmrt99B* is expressed as two domains in the ocular region (Fig. 6C-F). In *Parasteatoda*, first four domains of expression form in the head lobes (Fig. 6G), that shortly after become six (by splitting of the most posterior of the initial domains) (Fig. 6H-J). In *Euperipatoides*, two broad domains of expression are detectable in the head lobes (Fig. 6K-N). In all species, *Dmrt99B* is also expressed in the mouth at later developmental stages (asterisks in panels 6B, D, F, J, N). In the onychophoran, segmental expression appears at later stages in an anterior to posterior order that is likely associated with the formation of the openings of the nephridia (Fig. 6L-N) (cf. [58] Mayer 2006).

#### Doublesex (Dsx)

**Fig. 7:**
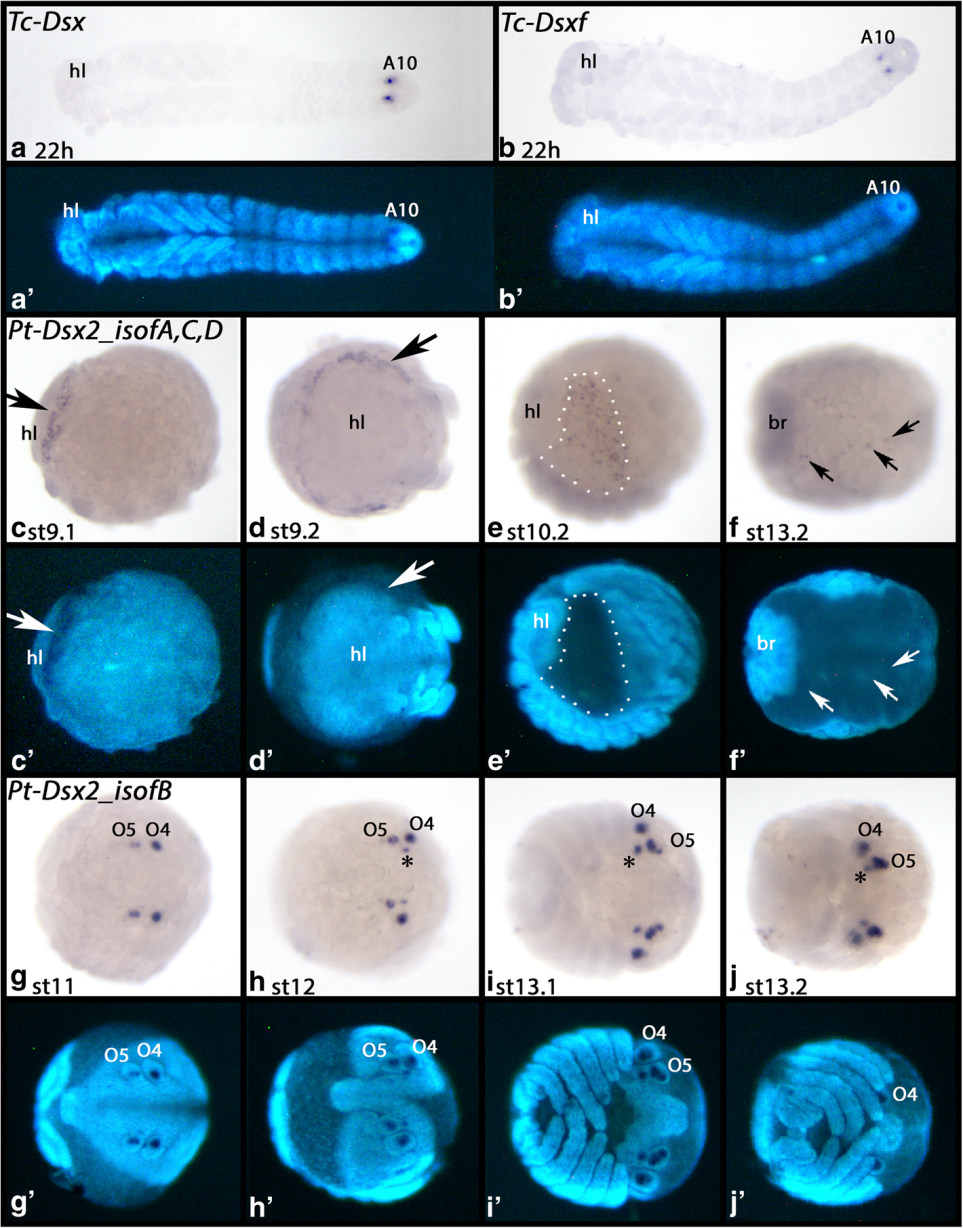
Expression of *Doublesex* (*Dsx*) in *Tribolium* (**A**, universal probe; **B**, female-specific probe. Note that the patterns appears strongly in half of the embryos, while it appears after prolonged staining time in the other half of the embryos. Figure 7B represents such latter case embryo.) and *Parasteatoda* (**C-F**, *Dsx2 isoformsA,C,D*; **G-J**, *Dsx2 isoformB*). Developmental stages are indicated. Panels A´-J´ represent DAPI stained embryos as seen in panels A-J. In all panels, anterior is to the left and ventral views, except panels C and E (lateral views) and F (dorsal view). Arrows in panel F point to single cells expressing the gene. Asterisks in panels H-J mark splitting expression in the fourth opisthosomal segment. Dashed lines in panel E/E´ mark the dorsal field. Abbreviations as in Fig. 4; A10, tenth abdominal segment; br, brain; O(x), opisthosomal segments. Note that the primordia of spider spinnerets are located on opisthosomal segments four (O4) and five (O5) (panels G-J)

From around 12 h after gastrulation onwards, *Tribolium Dsx* is first expressed in the form of a single domain in the tenth abdominal segment (Additional file 10: Figure S7A). Later this domain transforms into two dots (Fig. 7A/B and Additional file 10: Figure S7B/C). This pattern is present in all embryos hybridized with a universal probe detecting all isoforms of *Dsx* (Fig. 3b). The female-specific probe (targeting all female isoforms) (Figs. 3b and 7B) detected the same signal as the universal probe (Fig. 7A). However, in only approximately 50% of the embryos (20/37) the signal appears fast (within one hour) and equally strong as for the universal probe. In the other embryos, the same signal appears after an elongated staining period of at least 16 h, and the signal is significantly lower as for the general probe (Fig. 7B). We assume that these latter embryos represent males and that at least one of the “female-specific” isoforms of Dsx is expressed at low levels in male embryos as well.

We did not detect any specific signal of *Parasteatoda Dsx1* (*Pt-Dsx1*). The isoforms of *Pt-Dsx2*, however, are expressed in at least two unique patterns. A probe targeting isoforms A, C and D detects expression in a salt-and-pepper like pattern in the dorsal field surrounding the head lobes (Fig. 7C/D). At later stages, cells in the complete dorsal field express this isoform (Fig. 7E). In late developmental stages, the probe detects expression in a subset of cells on either side of the now closed dorsal midline (Fig. 7F). The *Pt-Dsx2* isoform B is exclusively expressed in the developing spinnerets, the silk-producing and processing organs of the spider (Fig. 7G-J). First, expression is in the form of single dots in the fourth and fifth opisthosomal segments (O4 and O5) (Fig. 7G). Later, additional small dot-like domains of expression appear in the morphologically differentiating spinnerets. At stage 12, this additional dot appears lateral to the expression in O5 (Fig. 7H). At stage 13.1, the initial expression in O5 splits into two (Fig. 7I).

We did not detect any specific signal of *Euperipatoides Dsx_like* (*Ek-Dsx_like*).

#### The orphan genes PtDmrt_like and PtDmrt_like2

**Fig. 8:**
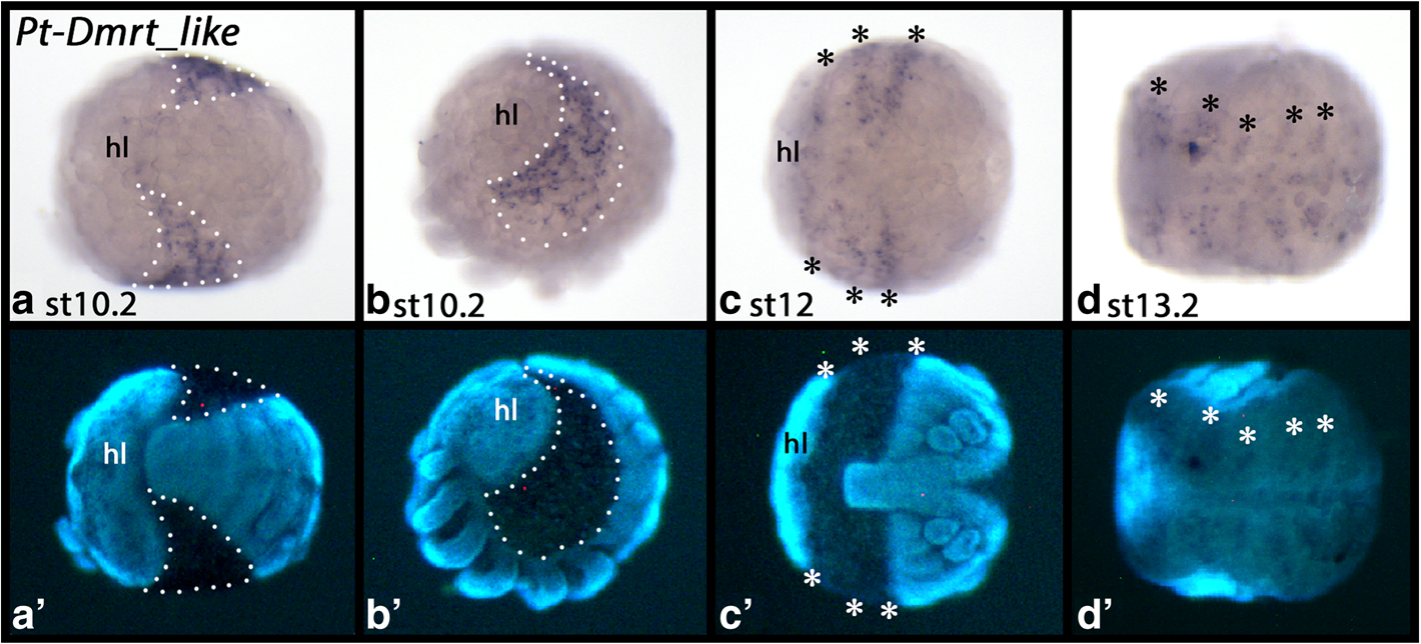
Expression of *Parasteatoda Dmrt_like*. Developmental stages are indicated. Panels A´-D´ represent DAPI stained embryos as seen in panels **A-D**. Panel A (ventral view), panel B (lateral view), panel C (posterior view), panel D (dorsal view). Dashed lines mark the dorsal field. Asterisks (*) mark metameric expression in the dorsal field. Abbreviations as in Fig. 4

Parasteatoda *Dmrt_like* is expressed in the dorsal field from approximately stage 10.2 onwards and throughout further development (Fig. 8). At later stages, a metameric pattern is seen within the dorsal field (Fig. 8C/D). We were not able to amplify *PtDmrt_like2* from cDNA synthesized from embryonic RNA.

## Discussion

### Gene expression suggests highly-conserved function of Dmrt genes

#### Dmrt11E

In both the onychophoran *Euperipatoides* and the spider *Parasteatoda*, *Dmrt11E* is expressed in the mesoderm of most of the appendages except for the frontal appendages of the former and the chelicerae of the latter (Fig. 4). In the myriapod *Glomeris*, however, *Dmrt11E* is only expressed in the mesoderm of the labrum and the mandibles, but not the other appendages. This raises the question if the (almost) universal function of Dmrt11E in the development of the mesoderm in the appendages has been lost in *Glomeris marginata*, myriapods in general, or even all Mandibulata. In *Tribolium*, this gene has been lost indicating that the function of *Dmrt11E* in mandibulates may be less critical than in other panarthropods. This is supported by the fact that *Dmrt11E* appears to be lost in the isopod *Armadillidium vulgare* as well ([17] Chebbi et al. 2019). Data from *Drosophila* are restricted to the published expression patterns on the BDGP in-situ homepage (Berkeley *Drosophila* Genome Project (see Additional file 3: Table S3 for the link: [59] Hammonds et al. 2013, [60] Tomancak et al. 2002, [61] Tomancak et al. 2007)). Here, the expression is described as ubiquitous with potential upregulation in the head and trunk mesoderm. Expression of *Dmrt11E* in the imaginal discs from which the appendages form in *Drosophila* has not been investigated. It appears, however, that *Dmrt11E* plays a role in the development of the limb-mesoderm, at least in non-mandibulate panarthropods. A role in mesoderm development could possibly be traced back to the last common ancestor of Bilateria since a similar function of Dmrt11E has been documented in vertebrates as well (the vertebrate ortholog is called *Dmrt2/Terra*) (e.g. [62] Meng et al. 1999, [63] Seo et al. 2006, [64] Sato et al. 2010). Unfortunately, none of the *Caenorhabditis elegans* Dmrt genes appears to represent a Dmrt11E ortholog ([5] Wexler et al. 2014), and data on ecdysozoan Dmrt11E outside Panarthropoda are not available. We isolated a *Dmrt93B* gene from embryonic cDNA of the priapulid *Priapulus caudatus*, but did not detect a specific embryonic expression pattern (note that gene expression studies in *Priapulus* are difficult and restricted to a short time window (e.g. [65] Martin-Duran et al. 2012)).

Likewise, there are no data on the embryonic expression of *Dmrt11E* in any spiralian species. The assumption that Dmrt11E genes play conserved function(s) in mesoderm patterning in Bilateria is thus solely based on data from panarthropods and vertebrates, two distantly related groups of animals.

#### Dmrt93B

Data on Dmrt93B genes is very limited, and functional investigations have only been undertaken in vertebrates (Dmrt3 gene group). There, the gene is inter alia expressed in the central nervous system (CNS), nasal placodes, Müllerian ducts, forming somites, and the developing gonads (e.g. [66] Smith et al. 2002, [67] Winkler et al. 2004, [68] Desmaris et al. 2018, [69] Yan et al. 2018).

Embryonic gene expression data or functional studies are not available from any protostomian animal, except for the model arthropod *Drosophila melanogaster*, and even here published data are restricted to the mere presentation of embryonic gene expression data on the BDGP in-situ homepage (Berkeley *Drosophila* Genome Project (see Additional file 3: Table S3 for the link)). Apart from that, the gene has also been investigated in the crustacean *Daphnia magna*, but in-situ gene expression data have not been provided ([12] Kato et al. 2008, [70] Kim et al. 2011).

In all the panarthropods investigated here, Dmrt93B orthologs are expressed in tissue associated with the developing mouth. In *Drosophila*, expression is found in the frontal ganglion, a structure that is associated with the stomatogastric nervous system (and thus with mouth development) ([71] Hartenstein 1997, [72] Ayali 2004). It is therefore likely that *Dmrt93B* genes play a conserved function in the development of the stomatogastric nervous system in Panarthropoda. The only information on Dmrt93B genes in another ecdysozoan comes from the nematode worm *Caenorhabditis elegans*. There the Dmrt93B gene *dmd-4* ([5] Wexler et al. 2014) is expressed in the head mesodermal cell (hmc), hypodermis, nervous system, and the head (WormBase, see Additional file 3: Table S3 for the link). Interestingly the hmc is located in the pseudo-coelom on the dorsal side of the pharynx ([73] Altun and Hall 2009). It is tempting to speculate that expression in the pharynx and stomatogastric nervous system in panarthropods and the expression in the hmc in the nematode could be evolutionary related. This would place the origin of the function of Dmrt93B/dmd-4 at the base of the Ecdysozoa (e.g. [55] Dunn et al. 2008). A very recent paper describes the expression of an uncharacterized Dmrt gene in a sea urchin which is exclusively expressed in the foregut ([74] Slota et al. 2019). We assume that this Dmrt gene is a likely ortholog of Dmrt93B which could place the origin of its function in mouth/foregut development at the base of the Bilateria. However, additional data from additional groups of animals are needed to corroborate this assumption.

#### Dmrt99B

*Dmrt99B* is expressed in the developing brain of all investigated panarthropod species (Fig. 6). Interestingly, the number of domains in which *Dmrt99B* is expressed in the brain seems to correspond with the number of eyes of the investigated organisms: two in *Euperipatoides* and *Glomeris*, and eight in *Parasteatoda* (and this correlation also appears to be conserved in *Drosophila* (Berkeley *Drosophila* Genome Project (see Additional file 3: Table S3 for the link))). In *Tribolium*, however, this correlation is absent. As for most beetles, *Tribolium* lacks ocelli and only possesses one pair of lateral eyes, but *Dmrt99B* is expressed in two additional domains in the brain (one per hemisphere). However, the ancestor of *Tribolium* likely possessed ocelli ([75] Leschen and Beutel 2004), and the additional domains of *Dmrt99B* expression in the head lobes of *Tribolium* may represent rudimentary structures associated with these ocelli. Interestingly, the bona fide arthropod eye markers *glass* (*gl*), *Pax6*, *dachshund* (*dac*), *eyes absent* (*eya*) and *sine oculis* (*so*) are expressed in similar (*gl*, *Pax6*, *eya*, *so*) or identical (*dac*) patterns as *Dmrt99B* ([76] Prpic and Tautz 2003, [77] Prpic 2005, [78] Liu and Friedrich 2004, [79] Yang et al. 2009a, [80] b). Likewise, other conserved markers of the optical field, like *scarecrow* (*scro/nkx2.1*), are expressed in conserved patterns in arthropods including *Drosophila*, *Tribolium* and *Glomeris* (Berkeley *Drosophila* Genome Project (see Additional file 3: Table S3 for the link), [81] Posnien et al. 2011, [82] Janssen 2017). In the spider *Parasteatoda*, the pattern of *Dmrt99B* very much resembles that of the combined eye-expressing genes ([83] Schomburg et al. 2015, see [84] Samadi et al. 2015 for a similar data set in another spider) suggesting an important and universal role in spider eye development. We assume that the expression domain marked with roman numeral 1 (Fig. 6G-J) may be connected to the development of the single pair of median eyes, and that the expression domains marked 2, 3a and 3b may be correlated to the formation of the three pairs of lateral eyes (cf. [83] Schomburg et al. 2015). However, a closer look at the correlation of optical systems and the expression of *Dmrt99B* suggests that its expression may not always be in the exact place where the eyes develop as it is the case in for example the onychophoran (Fig. 6M´). Expression of *Dmrt99B* may thus indeed rather be correlated with the regions of the brain that process vision than to the optical organs itself. Comparison with eye or brain-marker genes in species of the genus *Euperipatoides* reveals that the expression of *Dmrt99B* overlaps with many of these factors such as *orthodenticle* (*otd*), *scro/nkx2.1*, *retinal homeobox* (*rx*), *Pax6*, and *six3* ([85] Eriksson et al. 2013, [86] Franke et al. 2015, [82] Janssen 2017, [38] Janssen et al. 2018).

The compound eyes of arthropods are likely to represent an autapomorphy of Arthropoda, with the phylogenetically deepest examples being known from anomalocaridids ([87] Paterson et al. 2011) and the eyes of onychophorans thus likely have a different evolutionary origin (e.g. [88] Paulus 2000, [89] Bitsch and Bitsch 2005, [90] Mayer 2006). Therefore, it is not surprising that the eye-anlagen patterning genes such as *Dmrt99B* are not expressed in the onychophoran eye-anlagen.

In the nematode worm *Caenorhabditis*, the *Dmrt99B* ortholog *dmd-5* is expressed in the nervous system and the intestine (WormBase, see Additional file 3: Table S3 for the link).

Together our data strongly suggest that *Dmrt99B* orthologs are involved in the development of the eyes (and associated brain structures) in at least arthropods, but that its function in onychophorans is only in patterning brain structures that may be involved in processing visual information.

Another conserved aspect of *Dmrt99B* in Panarthropoda is its expression in the putative stomatogastric nervous system, similarly to the conserved expression of *Dmrt93B/Dmrt3* in these structures (discussed above). Interestingly, the vertebrate orthologs of *Dmrt99B*, *Dmrt4* and *Dmrt5*, act as neurogenic factors and, like *Dmrt93B/Dmrt3*, are involved in the development of the olfactory placodes (e.g. [48] Huang et al. 2005, [49] Parlier et al. 2013). If this expression in the stomatogastric nervous system and the olfactory placodes indeed represents conserved function, then this function must date back at least to the last common ancestor of all bilaterian animals, the Urbilateria.

### Potential sex-specific aspects of panarthropod Dmrt gene expression and isoform transcription

Dmrt genes are famous for their role in sex determination, gonad development, and the development of sex-specific morphological and behavioral traits (reviewed in e.g. [91] Kopp 2012 and [92] Picard et al. 2015). The most famous of these genes are represented by the *Drosophila Doublesex* (*Dsx*) gene ([93] Baker and Wolfner 1988), *mab-3* in *Caenorhabditis* ([26] Shen and Hodgkin 1988) and the Dmrt1 orthologs in vertebrates (e.g. [94] Nanda et al. 2002, [95] Matsuda et al. 2002) (discussed in the next section). However, even the other Dmrt genes often play roles in the development of the different sexes and sex-specific traits in various (but not all, e.g. [96] Reitzel et al. 2016) animal groups (e.g. [14] Zhang and Qiu 2010, [97] Johnsen and Andersen 2012, [98] Traylor-Knowles et al. 2015). In some cases, sex-specific differences are expressed through the activity of different isoforms of a given Dmrt gene ([3] Burtis and Baker 1989, [99] McKeown 1992, [100] Ottolenghi et al. 2000a, [101] b, [102] Ohbayashi et al. 2001, [10] Su et al. 2015). Differential gene expression of such isoforms has largely been investigated in adult tissues, rather than during development. In this study, we found one non-Dsx Dmrt gene that is spliced into (at least) two isoforms; this gene is *Glomeris Dmrt11E* (Fig. 3a). In *Glomeris*, the germ cell marker-gene *vasa* and Sox3 genes are all expressed in the mesoderm of the anal valves and in tissue anterior to that ([38] Janssen et al. 2018), and this is where *Dmrt11E* is (inter alia) expressed too (Fig. 4A-D, H). Additionally, *Gm-Dmrt11E* is expressed in the outer lining of the developing hindgut, tissue that potentially contributes to the developing gonads. Unfortunately, it was not possible to perform an isoform-specific in-situ hybridization for the shorter of the two *Dmrt11E* isoforms. Possibly, such in-situ experiment could uncover tissue- or tissue/sex-specific expression patterns (cf. expression of *Pt-Dsx2* isoforms (Fig. 7)).

For the other panarthropod non-Dsx Dmrt genes, there is no obvious correlation with gonadal development and/or differentiation of any other sex-specific trait, nor is any such gene expressed in a subset of embryos (a 50:50, or similar ratio) that would suggest sex-specific function already at such early stages of development. This, however, is not unexpected because sex-specific traits of panarthropods regularly develop after embryogenesis during post-embryonic or even post-larval developmental stages, or they are difficult to spot on morphological grounds. Examples include the initiation of sex-specific sexual behavior, the *Drosophila melanogaster* sex combs, the sex brushes of related drosophilids, the bulbi of male spiders, the development of the male phenotype in water fleas, the gonopods in male myriapods, and gonadal differentiation in general ([103] Coddington 1990, [104] Drago et al. 2008, [13] Kato et al. 2011, [105] Rice et al. 2018). In the future, it would be interesting to study expression of Dmrt genes in different sex-specific tissues of larval, juvenile and adult sex-specific structures in order to get a better understanding of the general role these genes may play in sexual differentiation.

### Doublesex

We could not detect expression in the investigated developmental stages for *Euperipatoides Dsx_like* and *Parasteatoda Dsx1*, and we did not detect a Dsx gene in the sequenced embryonic transcriptome of *Glomeris* (although Dsx genes have been identified in another myriapod, *Strigamia maritima* ([106] Chipman et al. 2014) (Fig. 1)), suggesting that these genes act later during development, and may then be involved in for example gonad development and differentiation.

At least one of the female-specific isoforms ([47] Shukla and Palli 2012, Fig. 3) of the single *Tribolium Dsx* gene, however, is expressed in a pattern that suggests a role in gonad development and/or differentiation (note that we could not use specific probes for the different female isoforms). At late embryonic developmental stages, *Tc-Dsx* is exclusively expressed in the form of two distinct dots in the tenth abdominal segment (Fig. 7A/B). Unfortunately, the universal germ line marker *vasa* is not expressed at these late developmental stages ([107] Schröder 2006), but another potential gonadal marker, *SoxE*, is expressed in the same segment as *Dsx* ([38] Janssen et al. 2018). Given that *Tribolium Dsx* is known to be involved in gonad development and sexual differentiation ([47] Shukla and Palli 2012), and that it is expressed in close proximity of *SoxE*, it is likely that the detected embryonic expression of *Dsx* is indeed in the developing gonads.

Interestingly, we found that at least one of the female-specific isoforms of *Tribolium* is expressed considerably stronger in about half of the investigated embryos, and we assume that these embryos represent females. In the other half of the embryos, the expression is much weaker. We assume that those embryos represent males in which at least one of the “female-specific” isoforms appear to be expressed albeit at a very low level (Additional file 10: Figure S7).

Sex-specific splicing of the sex-determining factor *Dsx* is common among insects, but not found in the crustacean *Daphnia magna* (and related cladocerans) implying that sex-specific splicing of Dsx could be an insect-specific trait ([47] Shukla and Palli 2012, [13] Kato et al. 2011, [53] Toyota et al. 2013, [108] Bopp et al. 2014). The fact that at least one of the two spider Dsx paralogs is also expressed in several isoforms raises the question if one (or more) of these isoforms could be sex-specific, and thus if sex-specific splicing of Dsx could be an ancestral feature of arthropod sex-determination. Interestingly, the different spider Dsx isoforms do not only differ outside their DM domain, but also contain different DM domains (Fig. 3c), which to our knowledge represents a unique feature of Dsx genes; at least in this detail the splice variants of insect Dsx and spider Dsx are fundamentally different.

At least one of the isoforms (A, C, D) of *Pt-Dsx2* is exclusively expressed in the dorsal field (DF) (also called extraembryonic area, layer, tissue or field ([109] Yamazaki et al. 2005, [110] Oda and Akiyama-Oda 2008, [111] Paese et al. 2018, [112] Hemmi et al. 2018)), first at the margin of the head lobes, later spreading over the complete DF (Fig. 7C-F). Similar expression in the DF has been reported for the GATA transcription factor *serpent* (*srp*) and *hepatocyte-nuclear-factor-4* (*hnf4*), and it has been suggested that these cells may contribute to yolk consumption and/or midgut development ([113] Feitosa et al. 2017 and references therein).

The isoform B of *Pt-Dsx*2 is exclusively expressed in the developing spinnerets. This is interesting for several reasons. First, spinnerets clearly represent an evolutionary novelty of spiders, and second because the set of spinnerets and their silk-producing glands is partially sex-specific ([114] Correa-Garhwal et al. 2017). *Pt-Dsx2* isoform B could thus, like so many other Dmrt genes in other animals, have been recruited for the establishment of sex-specific morphological differences in spiders. Evidence for this hypothesis comes from the work of [115] Schomburg (2017) who found that the same transcript of Dsx2 (*Dmrt1B* in his study) is indeed upregulated in developing male pedipalps (but not female pedipalps). The male pedipalps carry the spider copulation organs (bulbi) and thus display clear sex-specific morphological differences ([115] Schomburg 2017). Finally, the unique and distinct expression patterns of *Pt-Dsx2* isoform B vs the isoform(s) A, C and D strongly suggest that this gene has undergone multiple neo-functionalization processes. After the duplication into *Dsx1* and *Dsx2*, the latter likely acquired a new function during development (either in the DF or the spinnerets), and after that differential splicing allowed the gene to act independently in the development/differentiation of the other structure.

## Conclusions

Our data reveal that panarthropods possess a conserved set of Dmrt genes representing four classes: Dmrt11E, Dmrt93B, Dmrt99B and Doublesex (Dsx)/Dsx-like. The former three represent highly conserved factors with likely conserved functions dating back to the last common ancestor of arthropods and onychophorans. Dsx/Dsx-like Dmrt genes, however, which play important roles in animal sex determination and specification are much less conserved in their structure and embryonic expression profile reflecting their likely diverse function(s) in the differentiation of sex-specific traits.

## Additional files

Additional file 1: **Table S1.** Primer List. (XLSX 12 kb)

Additional file 2: **Table S2.** Accession numbers. (DOCX 61 kb)

Additional file 3: **Table S3.** Links. (DOCX 45 kb)

Additional file 4: **Figure S1.** Phylogenetic analysis based on the DM domain (with original branch length). Species abbreviations: Ek, *Euperipatoides kanangrensis*; Dm, *Drosophila melanogaster*; Gg, *Gallus gallus*; Gm, *Glomeris marginata*; Pc, *Priapulus caudatus*; Pt, *Parasteatoda tepidariorum*; Mm, *Mus musculus*; Sm, *Strigamia maritima*; Tc, *Tribolium castaneum*; Xt, *Xenopus tropicalis*. Green shade: Dmrt11E group. Red shade: Dmrt99B group (note that these genes form a monophyletic group in the phylogeny based on the complete ORFs (Fig. 1). Blue shade: Dmrt93B group. Yellow shade: Doublesex (Dsx) group. Magenta shade: orphan Dmrt genes. Node support is given as posterior probabilities. Open circles mark the two DM domains of *Pt-Dmrt_like2*. Asterisks mark the two different DM domains found in different splice variants of *Pt-Dsx2*. See text for further information. (TIF 43875 kb)

Additional file 5: **Figure S2.** Phylogenetic analysis based on DM domains. (see Additional file 4: Figure S1 for further information). (TIF 41433 kb)

Additional file 6: **Figure S3.** Phylogenetic analysis based on the complete ORFs (with original branch length). (see Fig. 1 for further information). (TIF 4446 kb)

Additional file 7: **Figure S4.** Alignment of DM domains. (TIF 33987 kb)

Additional file 8: **Figure S5.** Alignment of the complete ORFs. (TIF 35381 kb)

Additional file 9: **Figure S6.** Confocal data on the expression of *Euperipatoides Dmrt11E*. Magenta: Expression of *Dmrt11E*; Blue: DAPI. Panel A shows a Z-stack. Panels a1-a8 show a series of consecutive optical sections (6.5 μm per section) through part of the embryo. The focus is on the jaw-bearing segment (indicated by arrows). The data reveal that expression of *Dmrt11E* is exclusively inside the jaw, in mesodermal tissue, but not in the overlaying ectoderm. Abbreviations: fap, frontal appendage; j, jaw; L1, first walking-leg bearing segment; sp., slime papilla. (TIF 32533 kb)

Additional file 10: **Figure S7.** *Tribolium Dsxf* expression after 1 h of staining time. Orientation with anterior to the left in panels A and B. Variable orientation in panel C. After one hours of staining time, approximately half of the embryos incubated with the female specific probe of Dsx (*Dsxf*) stain (A, black arrow in C). The other embryos do not show any sign of expression (B, red circles in C). However, after prolonged staining time (> 16 h), these latter embryos also show expression in the same pattern as seen in the other embryos, albeit weaker (see Fig. 7b). (TIF 65562 kb)

## Data Availability

All data generated or analysed during this study are included in this published article and its Additional files.

## References

[1] Erdman SE, Burtis KC (1993). The *Drosophila doublesex* proteins share a novel zinc finger related DNA binding domain. EMBO J.

[2] Hildreth PE (1965). *Doublesex*, recessive gene that transforms both males and females of *Drosophila* into intersexes. Genetics.

[3] Burtis KC, Baker BS (1989). *Drosophila doublesex* gene controls somatic sexual differentiation by producing alternatively spliced mRNAs encoding related sex-specific polypeptides. Cell.

[4] Miller SW, Hayward DC, Bunch TA, Miller DJ, Ball EE, Bardwell VJ, Zarkower D, Brower DL (2003). A DM domain protein from a coral, *Acropora millepora*, homologous to proteins important for sex determination. Evol Dev.

[5] Wexler JR, Plachetzki DC, Kopp A (2014). Pan-metazoan phylogeny of the DMRT gene family: a framework for functional studies. Dev Genes Evol.

[6] Chen CJ, Shikina S, Chen WJ, Chung YJ, Chiu YL, Bertrand JA, Lee YH, Chang CF (2016). A novel female-specific and sexual reproduction-associated Dmrt gene discovered in the stony coral, *Euphyllia ancora*. Biol Reprod.

[7] Matsuda M, Shinomiya A, Kinoshita M (2007). DMY gene induces male development in genetically female (XX) medaka fish. Proc Natl Acad Sci U S A.

[8] Yoshimoto S, Okada E, Umemoto H, Tamura K, Uno Y, Nishida-Umehara C, Matsuda Y, Takamatsu N, Shiba T, Ito M (2008). A W-linked DM-domain gene, DM-W, participates in primary ovary development in *Xenopus laevis*. Proc Natl Acad Sci U S A.

[9] Matson CK, Murphy MW, Griswold MD, Yoshida S, Bardwell VJ, Zarkower D (2010). The mammalian *doublesex* homolog *DMRT1* is a transcriptional gatekeeper that controls the mitosis versus meiosis decision in male germ cells. Dev Cell.

[10] Su L, Zhou F, Ding Z, Gao Z, Wen J, Wei W, Wang Q, Wang W, Liu H (2015). Transcriptional variants of Dmrt1 and expression of four Dmrt genes in the blunt snout bream, *Megalobrama amblycephala*. Gene.

[11] Wang F, Yu Y, Ji D, Li H (2012). The DMRT gene family in amphioxus. J Biomol Struct Dyn.

[12] Kato Y, Kobayashi K, Oda S, Colbourn JK, Tatarazako N, Watanabe H, Iguchi T (2008). Molecular cloning and sexually dimorphic expression of DM-domain genes in *Daphnia magna*. Genomics.

[13] Kato Y, Kobayashi K, Watanabe H, Iguchi T (2011). Environmental sex determination in the branchiopod crustacean *Daphnia magna*: deep conservation of a *Doublesex* gene in the sex-determining pathway. PLoS Genet.

[14] Zhang EF, Qiu GF (2010). A novel Dmrt gene is specifically expressed in the testis of Chinese mitten crab, *Eriocheir sinensis*. Dev Genes Evol.

[15] Chandler JC, Fitzgibbon QP, Smith G, Elizur A, Ventura T (2017). Y-linked iDmrt1 paralogue (iDMY) in the eastern spiny lobster, *Sagmariasus verreauxi*: the first invertebrate sex-linked Dmrt. Dev Biol.

[16] Nong QD, Mohamad Ishak NS, Matsuura T, Kato Y, Watanabe H (2017). Mapping the expression of the sex determining factor *Doublesex1* in *Daphnia magna* using a knock-in reporter. Sci Rep.

[17] Chebbi Mohamed Amine, Becking Thomas, Moumen Bouziane, Giraud Isabelle, Gilbert Clément, Peccoud Jean, Cordaux Richard (2019). The Genome of Armadillidium vulgare (Crustacea, Isopoda) Provides Insights into Sex Chromosome Evolution in the Context of Cytoplasmic Sex Determination. Molecular Biology and Evolution.

[18] Oliveira DC, Werren JH, Verhulst EC, Giebel JD, Kamping A, Beukeboom LW, van de Zande L (2009). Identification and characterization of the doublesex gene of *Nasonia*. Insect Mol Biol.

[19] Kijimoto T, Moczek AP, Andrews J (2012). Diversification of *doublesex* function underlies morph-, sex-, and species-specific development of beetle horns. Proc Natl Acad Sci U S A.

[20] Gotoh H, Miyakawa H, Ishikawa A, Ishikawa Y, Sugime Y, Emlen DJ, Lavine LC, Miura T (2014). Developmental link between sex and nutrition; *doublesex* regulates sex-specific mandible growth via juvenile hormone signaling in stag beetles. PLoS Genet.

[21] Gotoh H, Zinna RA, Warren I (2016). Identification and functional analyses of sex determination genes in the sexually dimorphic stag beetle *Cyclommatus metallifer*. BMC Genomics.

[22] Komata S, Lin CP, Iijima T, Fujiwara H, Sota T (2016). Identification of *doublesex* alleles associated with the female-limited Batesian mimicry polymorphism in *Papilio memnon*. Sci Rep.

[23] Price DC, Egizi A, Fonseca DM (2015). The ubiquity and ancestry of insect *doublesex*. Sci Rep.

[24] Xu J, Zhan S, Chen S, Zeng B, Li Z, James AA, Tan A, Huang Y (2017). Sexually dimorphic traits in the silkworm, *Bombyx mori*, are regulated by doublesex. Insect Biochem Mol Biol.

[25] Chong T, Collins JJ, Brubacher JL, Zarkower D, Newmark PA (2013). A sex-specific transcription factor controls male identity in a simultaneous hermaphrodite. Nat Commun.

[26] Shen MM, Hodgkin J (1988). *mab-3*, a gene required for sex-specific yolk protein expression and a male-specific lineage in *C. elegans*. Cell.

[27] Mason DA, Rabinowitz JS, Portman DS (2008). *dmd-3*, a *doublesex*-related gene regulated by *tra-1*, governs sex-specific morphogenesis in *C. elegans*. Development.

[28] Siehr MS, Koo PK, Sherlekar AL, Bian X, Bunkers MR, Miller RM, Portman DS, Lints R (2011). Multiple *doublesex*-related genes specify critical cell fates in a *C. elegans* male neural circuit. PLoS one.

[29] Morrow JL, Riegler M, Frommer M, Shearman DC (2014). Expression patterns of sex-determination genes in single male and female embryos of two *Bactrocera* fruit fly species during early development. Insect Mol Biol.

[30] Grossmann D, Prpic NM (2012). Egfr signaling regulates distal as well as medial fate in the embryonic leg of *Tribolium castaneum*. Dev Biol.

[31] Janssen R, Prpic NM, Damen WG (2004). Gene expression suggests decoupled dorsal and ventral segmentation in the millipede *Glomeris marginata* (Myriapoda: Diplopoda). Dev Biol.

[32] Prpic N.-M., Schoppmeier M., Damen W. G.M. (2008). Collection and Fixation of Spider Embryos. Cold Spring Harbor Protocols.

[33] Hogvall M, Schönauer A, Budd GE, McGregor AP, Posnien N, Janssen R (2014). Analysis of the Wnt gene repertoire in an onychophoran provides new insights into the evolution of segmentation. Evodevo.

[34] Strobl F, Stelzer EH (2014). Non-invasive long-term fluorescence live imaging of *Tribolium castaneum* embryos. Development.

[35] Mittmann B, Wolff C (2012). Embryonic development and staging of the cobweb spider *Parasteatoda tepidariorum* C. L. Koch, 1841 (syn.: *Achaearanea tepidariorum*; Araneomorphae; Theridiidae). Dev Genes Evol.

[36] Janssen R, Budd GE (2013). Deciphering the onychophoran 'segmentation gene cascade': gene expression reveals limited involvement of pair rule gene orthologs in segmentation, but a highly conserved segment polarity gene network. Dev Biol.

[37] Janssen R, Posnien N (2014). Identification and embryonic expression of *Wnt2*, *Wnt4*, *Wnt5* and *Wnt9* in the millipede *Glomeris marginata* (Myriapoda: Diplopoda). Gene Expr Patterns.

[38] Janssen R, Andersson E, Betner E, Bijl S, Fowler W, Höök L, Lehr J, Mannelqvist A, Panara V, Smith K, Tieman S (2018). Embryonic expression patterns and phylogenetic analysis of panarthropod sox genes: insight into nervous system development, segmentation and gonadogenesis. BMC Evol Biol.

[39] Notredame C, Higgins DG, Heringa J (2000). T-coffee: a novel method for fast and accurate multiple sequence alignment. J Mol Biol.

[40] Gouy M, Guindon S, Gascuel O (2010). SeaView version 4: a multiplatform graphical user interface for sequence alignment and phylogenetic tree building. Mol Biol Evol.

[41] Larsson A (2014). AliView: a fast and lightweight alignment viewer and editor for large data sets. Bioinformatics.

[42] Huelsenbeck JP, Ronquist F (2001). MRBAYES: bayesian inference of phylogenetic trees. Bioinformatics.

[43] Kimball S, Mattis P, Natterer M. The GIMP Team (2018) GNU image manipulation program V.2.10.0. The GIMP team URL: www.gimp.org

[44] Ottolenghi C, Fellous M, Barbieri M, McElreavey K (2002). Novel paralogy relations among human chromosomes support a link between the phylogeny of doublesex-related genes and the evolution of sex determination. Genomics.

[45] Pomerantz AF, Hoy MA, Kawahara AY (2015). Molecular characterization and evolutionary insights into potential sex-determination genes in the western orchard predatory mite *Metaseiulus occidentalis* (Chelicerata: Arachnida: Acari: Phytoseiidae). J Biomol Struct Dyn.

[46] Jia LY, Chen L, Keller L, Wang J, Xiao JH, Huang DW (2018). Doublesex evolution is correlated with social complexity in ants. Genome Biol Evol.

[47] Shukla JN, Palli S (2012). Doublesex target genes in the red flour beetle, *Tribolium castaneum*. Sci Rep.

[48] Huang X, Hong C, O’Donnel M, Saint-Jeannet JP (2005). The *doublesex*-related gene, *XDmrt4*, is required for neurogenesis in the olfactory system. Proc Natl Acad Sci U S A.

[49] Parlier D, Moers V, Van Campenhout C, Preillon J, Leclère L, Saulnier A, Sirakov M, Busengdal H, Kricha S, Marine JC, Rentzsch F, Bellefroid EJ (2013). The *Xenopus doublesex*-related gene *Dmrt5* is required for olfactory placode neurogenesis. Dev Biol.

[50] Volff JN, Zarkower D, Bardwell VJ, Schartl M (2003). Evolutionary dynamics of the DM domain gene family in metazoans. J Mol Evol.

[51] An W, Cho S, Ishii H, Wensink PC (1996). Sex-specific and non-sex-specific oligomerization domains in both of the *doublesex* transcription factors from *Drosophila melanogaster*. Mol Cell Biol.

[52] Bayrer JR, Zhang W, Weiss MA (2005). Dimerization of doublesex is mediated by a cryptic ubiquitin-associated domain fold: implications for sex-specific gene regulation. J Biol Chem.

[53] Toyota K, Kato Y, Sato M, Sugiura N, Miyagawa S, Miyakawa H, Watanabe H, Oda S, Ogino Y, Hiruta C, Mizutani T, Tatarazako N, Paland S, Jackson C, Colbourne JK, Iguchi T (2013). Molecular cloning of *doublesex* genes of four cladocera (water flea) species. BMC Genomics.

[54] Webster BL, Copley RR, Jenner RA, Mackenzie-Dodds JA, Bourlat SJ, Rota-Stabelli O, Littlewood DT, Telford MJ (2006). Mitogenomics and phylogenomics reveal priapulid worms as extant models of the ancestral Ecdysozoan. Evol Dev.

[55] Dunn CW, Hejnol A, Matus DQ, Pang K, Browne WE, Smith SA, Seaver E, Rouse GW, Obst M, Edgecombe GD, Sorensen MV, Haddock SH, Schmidt-Rhaesa A, Okusu A, Kristensen RM, Wheeler WC, Martindale MQ, Giribet G (2008). Broad phylogenomic sampling improves resolution of the animal tree of life. Nature.

[56] Gunalan K, Gao X, Yap SS, Huang X, Preiser PR (2013). The role of the reticulocyte-binding-like protein homologues of *Plasmodium* in erythrocyte sensing and invasion. Cell Microbiol.

[57] Marchler-Bauer A, Bo Y, Han L, He J, Lanczycki CJ, Lu S, Chitsaz F, Derbyshire MK, Geer RC, Gonzales NR, Gwadz M, Hurwitz DI, Lu F, Marchler GH, Song JS, Thanki N, Wang Z, Yamashita RA, Zhang D, Zheng C, Geer LY, Bryant SH (2017). CDD/SPARCLE: functional classification of proteins via subfamily domain architectures. Nucleic Acids Res.

[58] Mayer G (2006). Origin and differentiation of nephridia in the Onychophora provide no support for the Articulata. Zoomorphology.

[59] Hammonds AS, Bristow CA, Fisher WW, Weiszmann R, Wu S, Hartenstein V, Kellis M, Yu B, Frise E, Celniker SE (2013). Spatial expression of transcription factors in *Drosophila* embryonic organ development. Genome Biol.

[60] Tomancak P, Beaton A, Weiszmann R, Kwan E, Shu S, Lewis SE, Richards S, Ashburner M, Hartenstein V, Celniker SE, Rubin GM (2002). Systematic determination of patterns of gene expression during *Drosophila* embryogenesis. Genome Biol.

[61] Tomancak P, Berman BP, Beaton A, Weiszmann R, Kwan E, Hartenstein V, Celniker SE, Rubin GM (2007). Global analysis of patterns of gene expression during *Drosophila* embryogenesis. Genome Biol.

[62] Meng A, Moore B, Tang H, Yuan B, Lin S (1999). A *Drosophila doublesex*-related gene, *terra*, is involved in somitogenesis in vertebrates. Development.

[63] Seo KW, Wang Y, Kokubo H, Kettlewell JR, Zarkower DA, Johnson RL (2006). Targeted disruption of the DM domain containing transcription factor Dmrt2 reveals an essential role in somite patterning. Dev Biol.

[64] Sato T, Rocancourt D, Marques L, Thorsteinsdóttir S, Buckingham M (2010). A Pax3/Dmrt2/Myf5 regulatory cascade functions at the onset of myogenesis. PLoS Genet.

[65] Martín-Durán JM, Janssen R, Wennberg S, Budd GE, Hejnol A (2012). Deuterostomic development in the protostome *Priapulus caudatus*. Curr Biol.

[66] Smith CA, Hurley TM, McClive PJ, Sinclair AH (2002). Restricted expression of DMRT3 in chicken and mouse embryos. Mech Dev.

[67] Winkler C, Hornung U, Kondo M, Neuner C, Duschl J, Shima A, Schartl M (2004). Developmentally regulated and non-sex-specific expression of autosomal dmrt genes in embryos of the Medaka fish (*Oryzias latipes*). Mech Dev.

[68] Desmaris Elodie, Keruzore Marc, Saulnier Amandine, Ratié Leslie, Assimacopoulos Stavroula, De Clercq Sarah, Nan Xinsheng, Roychoudhury Kaushik, Qin Shenyue, Kricha Sadia, Chevalier Clément, Lingner Thomas, Henningfeld Kristine A., Zarkower David, Mallamaci Antonello, Theil Thomas, Campbell Kenneth, Pieler Tomas, Li Meng, Grove Elizabeth A., Bellefroid Eric J. (2018). DMRT5, DMRT3, and EMX2 Cooperatively Repress Gsx2 at the Pallium–Subpallium Boundary to Maintain Cortical Identity in Dorsal Telencephalic Progenitors. The Journal of Neuroscience.

[69] Yan H, Shen X, Cui X, Wu Y, Wang L, Zhang L, Liu Q, Jiang Y (2018). Identification of genes involved in gonadal sex differentiation and the dimorphic expression pattern in *Takifugu rubripes* gonad at the early stage of sex differentiation. Fish Physiol Biochem.

[70] Kim J, Kim Y, Lee S, Kwak K, Chung WJ, Choi K (2011). Determination of mRNA expression of DMRT93B, vitellogenin, and cuticle 12 in *Daphnia magna* and their biomarker potential for endocrine disruption. Ecotoxicology.

[71] Hartenstein V (1997). Development of the insect stomatogastric nervous system. Trends Neurosci.

[72] Ayali A (2004). The insect frontal ganglion and stomatogastric pattern generator networks. Neurosignals.

[73] Altun ZF, Hall DH (2009) Muscle system, head mesodermal cell. In WormAtlas. 10.3908/wormatlas.1.10 edited for the web by Laura a. Herndon. Last revision: June 1, 2012.

[74] Slota LA, Miranda EM, McClay DR (2019). Spatial and temporal patterns of gene expression during neurogenesis in the sea urchin *Lytechinus variegatus*. Evodevo.

[75] Leschen RAB, Beutel RG (2004). Ocellar atavism in Coleoptera: Plesiomorphy or apomorphy?. J Zool Syst Evol Res.

[76] Prpic NM, Tautz D (2003). The expression of the proximodistal axis patterning genes *Distal-less* and *dachshund* in the appendages of *Glomeris marginata* (Myriapoda: Diplopoda) suggests a special role of these genes in patterning the head appendages. Dev Biol.

[77] Prpic NM (2005). Duplicated *Pax6* genes in *Glomeris marginata* (Myriapoda: Diplopoda), an arthropod with simple lateral eyes. Zoology.

[78] Liu Z, Friedrich M (2004). The *Tribolium* homologue of *glass* and the evolution of insect larval eyes. Dev Biol.

[79] Yang X, Zarinkamar N, Bao R, Friedrich M (2009). Probing the *Drosophila* retinal determination gene network in *Tribolium* (I): the early retinal genes *dachshund*, *eyes absent* and *sine oculis*. Dev Biol.

[80] Yang X, Weber M, Zarinkamar N, Posnien N, Friedrich F, Wigand B, Beutel R, Damen WG, Bucher G, Klingler M, Friedrich M (2009). Probing the *Drosophila* retinal determination gene network in *Tribolium* (II): the Pax6 genes *eyeless* and *twin of eyeless*. Dev Biol.

[81] Posnien N, Koniszewski ND, Hein HJ, Bucher G (2011). Candidate gene screen in the red flour beetle *Tribolium* reveals *six3* as ancient regulator of anterior median head and central complex development. PLoS Genet.

[82] Janssen R (2017). Comparative analysis of gene expression patterns in the arthropod labrum and the onychophoran frontal appendages, and its implications for the arthropod head problem. Evodevo.

[83] Schomburg C, Turetzek N, Schacht MI, Schneider J, Kirfel P, Prpic NM, Posnien N (2015). Molecular characterization and embryonic origin of the eyes in the common house spider *Parasteatoda tepidariorum*. Evodevo.

[84] Samadi L, Schmid A, Eriksson BJ (2015). Differential expression of retinal determination genes in the principal and secondary eyes of *Cupiennius salei* Keyserling (1877). Evodevo.

[85] Eriksson BJ, Samadi L, Schmid A (2013). The expression pattern of the genes *engrailed*, *pax6*, *otd* and *six3* with special respect to head and eye development in *Euperipatoides kanangrensis* Reid 1996 (Onychophora: Peripatopsidae). Dev Genes Evol.

[86] Franke FA, Schumann I, Hering L, Mayer G (2015). Phylogenetic analysis and expression patterns of Pax genes in the onychophoran *Euperipatoides rowelli* reveal a novel bilaterian Pax subfamily. Evol Dev.

[87] Paterson JR, García-Bellido DC, Lee MS, Brock GA, Jago JB, Edgecombe GD (2011). Acute vision in the giant Cambrian predator Anomalocaris and the origin of compound eyes. Nature.

[88] Paulus HF (2000). Phylogeny of the Myriapoda. Crustacea, Insecta: a new attempt using photoreceptor structure. J Zool Syst Evol Res.

[89] Bitsch C, Bitsch J, Koenemann S (2005). Evolution of eye structure and arthropod phylogeny. Crustaceans and arthropod relationships, CRC press.

[90] Mayer G (2006). Structure and development of onychophoran eyes: what is the ancestral visual organ in arthropods?. Arthropod Struct Dev.

[91] Kopp A (2012). Dmrt genes in the development and evolution of sexual dimorphism. Trends Genet.

[92] Picard MA, Cosseau C, Mouahid G, Duval D, Grunau C, Toulza È, Allienne JF, Boissier J (2015). The roles of Dmrt (double sex/male-abnormal-3 related transcription factor) genes in sex determination and differentiation mechanisms: ubiquity and diversity across the animal kingdom. C R Biol.

[93] Baker BS, Wolfner MF (1988). A molecular analysis of *doublesex*, a bifunctional gene that controls both male and female sexual differentiation in *Drosophila melanogaster*. Genes Dev.

[94] Nanda I, Kondo M, Hornung U, Asakawa S, Winkler C, Shimizu A, Shan Z, Haaf T, Shimizu N, Shima A, Schmid M, Schartl M (2002). A duplicated copy of DMRT1 in the sex-determining region of the Y chromosome of the medaka, *Oryzias latipes*. Proc Natl Acad Sci U S A.

[95] Matsuda M, Nagahama Y, Shinomiya A, Sato T, Matsuda C, Kobayashi T, Morrey CE, Shibata N, Asakawa S, Shimizu N, Hori H, Hamaguchi S, Sakaizumi M (2002). DMY is a Y-specific DM-domain gene required for male development in the medaka fish. Nature.

[96] Reitzel AM, Pang K, Martindale MQ (2016). Developmental expression of "germline"- and "sex determination"-related genes in the ctenophore *Mnemiopsis leidyi*. Evodevo.

[97] Johnsen H, Andersen Ø (2012). Sex dimorphic expression of five dmrt genes identified in the Atlantic cod genome. The fish-specific dmrt2b diverged from dmrt2a before the fish whole-genome duplication. Gene.

[98] Traylor-Knowles NG, Kane EG, Sombatsaphay V, Finnerty JR, Reitzel AM (2015). Sex-specific and developmental expression of Dmrt genes in the starlet sea anemone, *Nematostella vectensis*. Evodevo.

[99] McKeown M (1992). Sex differentiation: the role of alternative splicing. Curr Opin Genet Dev.

[100] Ottolenghi C, Veitia R, Quintana-Murci L, Torchard D, Scapoli L, Souleyreau-Therville N, Beckmann J, Fellous M, McElreavey K (2000). The region on 9p associated with 46, XY sex reversal contains several transcripts expressed in the urogenital system and a novel doublesex-related domain. Genomics..

[101] Ohbayashi F, Suzuki MG, Mita K, Okano K, Shimada T (2001). A homologue of th *Drosophila doublesex* gene is transcribed into sex-specific mRNA isoforms in the silkworm, *Bombyx mori*. Comp Biochem Physiol B Biochem Mol Biol.

[102] Ottolenghi C, Veitia R, Barbieri M, Fellous M, McElreavey K (2000). The human *doublesex*-related gene, DMRT2, is homologous to a gene involved in somitogenesis and encodes a potential bicistronic transcript. Genomics..

[103] Coddington J (1990). Ontogeny and homology in the male palpus of orb weaving spiders and their relatives, with comments on phylogeny (Araneoclada: Araneoidea, Deinopoidea). Smithson Contrib to Zool.

[104] Drago L, Fusco G, Minelli A (2008). Non-systemic metamorphosis in male millipede appendages: long delayed, reversible effect of an early localized positional marker?. Front Zool.

[105] Rice G, Barmina O, Hu K, Kopp A (2018). Evolving *doublesex* expression correlates with the origin and diversification of male sexual ornaments in the *Drosophila immigrans* species group. Evol Dev.

[106] Chipman AD, Ferrier DE, Brena C (2014). The first myriapod genome sequence reveals conservative arthropod gene content and genome organisation in the centipede *Strigamia maritima*. PLoS Biol.

[107] Schröder R (2006). *vasa* mRNA accumulates at the posterior pole during blastoderm formation in the flour beetle *Tribolium castaneum*. Dev Genes Evol.

[108] Bopp D, Saccone G, Beye M (2014). Sex determination in insects: variations on a common theme. Sex Dev.

[109] Yamazaki K, Akiyama-Oda Y, Oda H (2005). Expression patterns of a *twist*-related gene in embryos of the spider *Achaearanea tepidariorum* reveal divergent aspects of mesoderm development in the fly and spider. Zool Sci.

[110] Oda H, Akiyama-Oda Y (2008). Differing strategies for forming the arthropod body plan: lessons from Dpp, Sog and Delta in the fly *Drosophila* and spider *Achaearanea*. Develop Growth Differ.

[111] Paese CLB, Schoenauer A, Leite DJ, Russell S, McGregor AP (2018). A SoxB gene acts as an anterior gap gene and regulates posterior segment addition in a spider. Elife.

[112] Hemmi N, Akiyama-Oda Y, Fujimoto K, Oda H (2018). A quantitative study of the diversity of stripe-forming processes in an arthropod cell-based field undergoing axis formation and growth. Dev Biol.

[113] Feitosa NM, Pechmann M, Schwager EE, Tobias-Santos V, McGregor AP, Damen WGM, Nunes da Fonseca R (2017). Molecular control of gut formation in the spider *Parasteatoda tepidariorum*. Genesis.

[114] Correa-Garhwal SM, Chaw RC, Clarke TH, Ayoub NA, Hayashi CY (2017). Silk gene expression of theridiid spiders: implications for male-specific silk use. Zoology (Jena).

[115] Schomburg C (2017) Developmental Studies on Eye Types and Pedipalps in *Parasteatoda tepidariorum*. PhD Thesis. Georg-August-Universiät Göttingen, Göttingen, Germany. https://ediss.uni-goettingen.de/handle/11858/00-1735-0000-002E-E304-3.

