# Supplementary material for: Phylogenetic analysis and embryonic expression of panarthropod Dmrt genes
Source: Front Zool. 2019 Jul 2;16:23. doi: 10.1186/s12983-019-0322-0 (PMC6604209; doi:10.1186/s12983-019-0322-0)
Supplement: Supplementary file 2 — Table S2. Accession numbers. (DOCX 61 kb) [file 12983_2019_322_MOESM2_ESM.docx]

*Drosophila melanogaster*

DmDmrt11E NM_078591.3

DmDmrt93B NP_524428.1

DmDmrt99B NP_524549.1

DmDsx AAA17840.1

*Daphnia magna*

DmaDmrt11E AB361069

DmaDmrt93B AB361070

DmaDmrt99B AB361071

DmaDsx AB569296, AB569297, AB569298.

*Euperipatoides kanangrensis*

Ek-Dmrt11E LR027813

EkDmrt93B LR027814

EkDmrt99B LR027815

EkDsx_like LR027816

*Gallus gallus*

GgDmrt1 Q9PTQ7.2

GgDmrt2 XP_003643035.3

GgDmrt3 XP_429193.2

GgDmrtA2 XP_015146712.1

GgDmrtB1 NP_001232910.1

*Glomeris marginata*

GmDmrt11E LR027810

GmDmrt93B LR027811

GmDmrt99B LR027812

*Mus musculus*

MmDmrt1 NP_056641.2

MmDmrt2 NP_665830.1

MmDmrt3 NP_796334.2

MmDmrtA1 NP_783578.1

MmDmrtA2 NP_758500.2

MmDmrtB1 NP_063925.1

MmDmrtC2 XP_017167796.1

*Priapulus caudatus*

PcDmrt11E XM_014820073.1

PcDmrt93B XM_014819542.1

PcDmrt99B XM_014810247.1

*Parasteatoda tepidariorum*

PtDmrt11E XM_016068462.1

PtDmrt93B XM_016071185.1

PtDmrt99B XM_016071184.1

PtDsx1 XM_021148633.1

PtDsx2A XM_021148637.1

PtDsx2B XM_021148638.1

PtDsx2C XM_021148639.1

PtDmrt_like XM_021148640.1

PtDmrt_like2 XM_016057845.1

*Strigamia maritima*

SmDmrt93B Strigamia_SMAR010523

SmDmrt11E Strigamia_SMAR010498

SmDmrt99B Strigamia_SMAR010524

SmDsx1 Strigamia_SMAR006330

SmDsx2 Strigamia_SMAR002496

*Tribolium castaneum*

TcDmrt93B Tc_EFA12115_1

TcDsxM XM_001807396.3

TcDsxF JQ857099.1, JQ857100.1, JQ857101.1

TcDmrt99B Tc_XP_975675_1

*Xenopus tropicalis*

XtDmrt1 P85119.1

XtDmrt2 NP_001093726.1

XtDmrt3 NP_001243149.1

XtDmrtA1 NP_001072447.1

XtDmrtA2 NP_001096543.1

XtDmrtB1 XP_002931473.1
