# Supplementary material for: Phylogenetic analysis and embryonic expression of panarthropod Dmrt genes
Source: Front Zool. 2019 Jul 2;16:23. doi: 10.1186/s12983-019-0322-0 (PMC6604209; doi:10.1186/s12983-019-0322-0)
Supplement: Supplementary file 3 — Table S3. Links. (DOCX 45 kb) [file 12983_2019_322_MOESM3_ESM.docx]

| Species and Gene Name | Link |
| --- | --- |
| *Drosophila Dmrt11E* | http://insitu.fruitfly.org/cgi-bin/ex/search.pl?ftype=0&ftext=Dmrt11E |
| *Drosophila Dmrt93B* | http://insitu.fruitfly.org/cgi-bin/ex/search.pl?ftype=0&ftext=Dmrt93B |
| *Drosophila Dmrt99B* | http://insitu.fruitfly.org/cgi-bin/ex/search.pl?ftype=0&ftext=Dmrt99B |
| *Drosophila scarecrow* | http://insitu.fruitfly.org/cgi-bin/ex/search.pl?ftype=0&ftext=scro |
| *Caenorhabditis dmd-5* | https://wormbase.org/search/gene/F10C1.5?from=https://wormbase.org/db/gene/gene?name=F10C1.5;class=Gene |
| *Caenorhabditis*  *dmd-4* | https://www.wormbase.org/search/all/gene%20dmd-4?redirect=1 |
