# Supplementary figures and images for: Phylogenetic analysis and embryonic expression of panarthropod Dmrt genes
Source: Front Zool. 2019 Jul 2;16:23. doi: 10.1186/s12983-019-0322-0 (PMC6604209; doi:10.1186/s12983-019-0322-0)

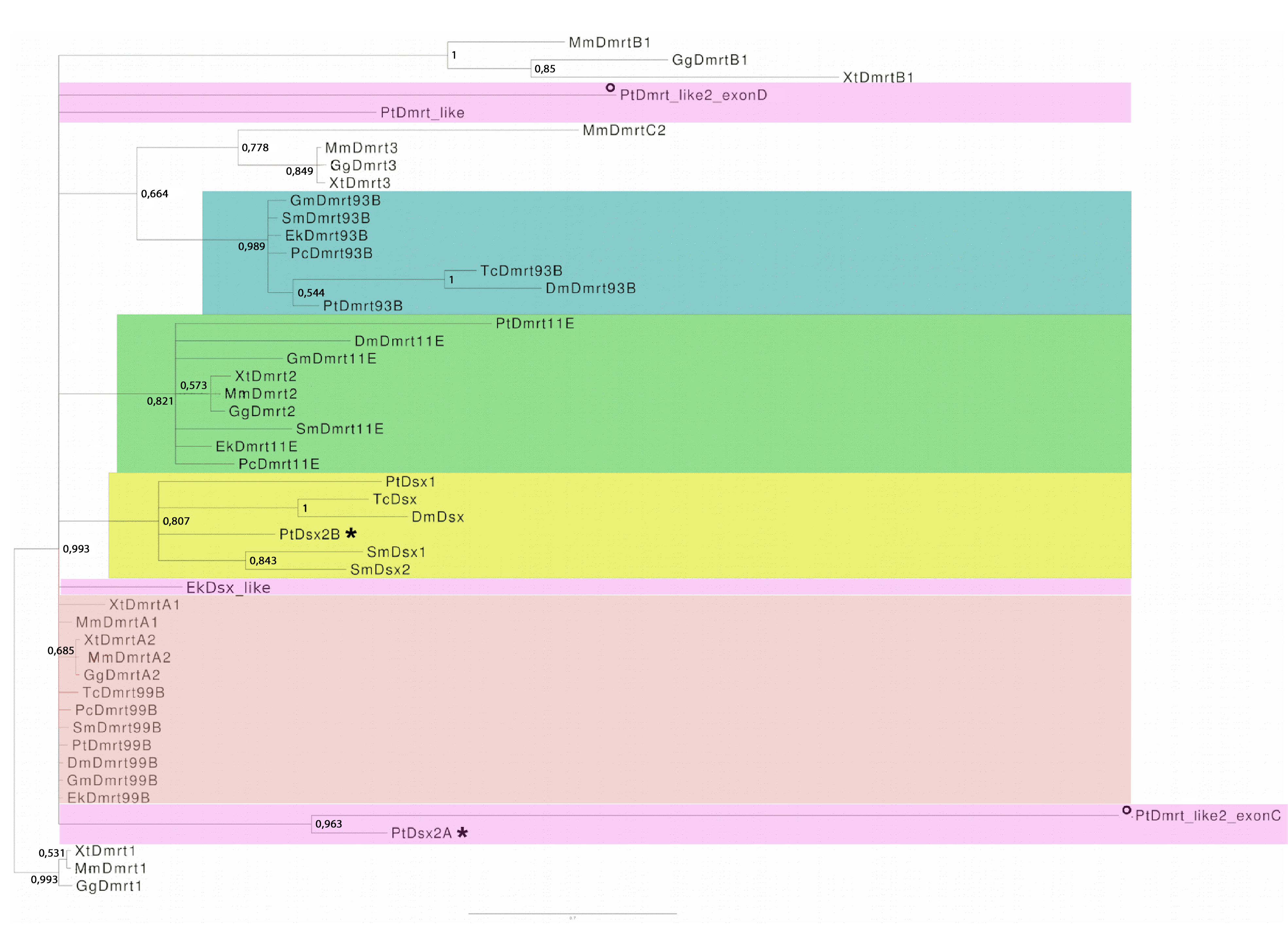

Supplement: Supplementary file 4 — Figure S1. Phylogenetic analysis based on the DM domain (with original branch length). Species abbreviations: Ek, Euperipatoides kanangrensis; Dm, Drosophila melanogaster; Gg, Gallus gallus; Gm, Glomeris marginata; Pc, Priapulus caudatus; Pt, Parasteatoda tepidariorum; Mm, Mus musculus; Sm, Strigamia maritima; Tc, Tribolium castaneum; Xt, Xenopus tropicalis. Green shade: Dmrt11E group. Red shade: Dmrt99B group (note that these genes form a monophyletic group in the phylogeny based on the complete ORFs (Fig. 1). Blue shade: Dmrt93B group. Yellow shade: Doublesex (Dsx) group. Magenta shade: orphan Dmrt genes. Node support is given as posterior probabilities. Open circles mark the two DM domains of Pt-Dmrt_like2. Asterisks mark the two different DM domains found in different splice variants of Pt-Dsx2. See text for further information. (TIF 43875 kb) [file 12983_2019_322_MOESM4_ESM.tif]

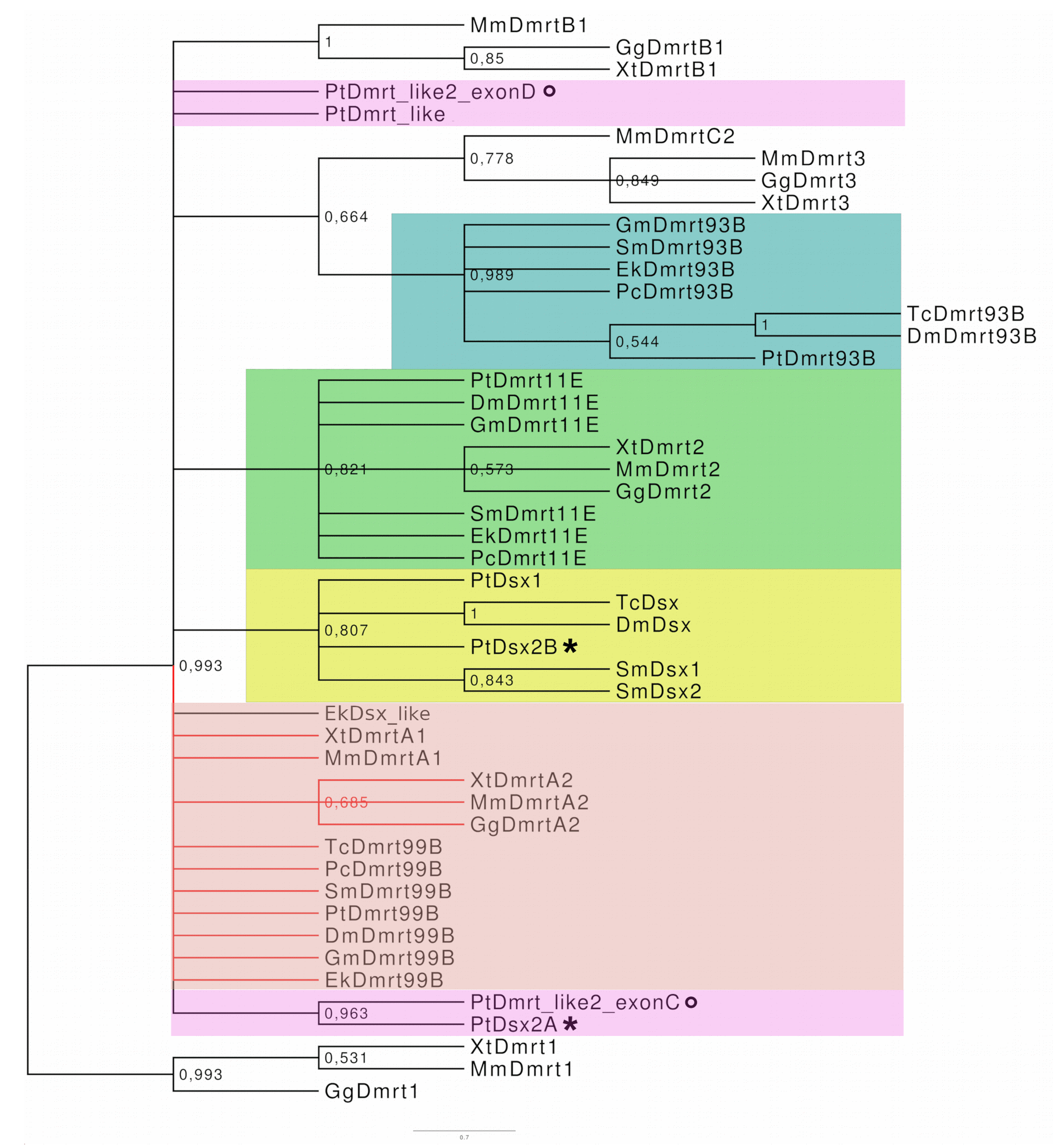

Supplement: Supplementary file 5 — Figure S2. Phylogenetic analysis based on DM domains. (see Additional file 4: Figure S1 for further information). (TIF 41433 kb) [file 12983_2019_322_MOESM5_ESM.tif]

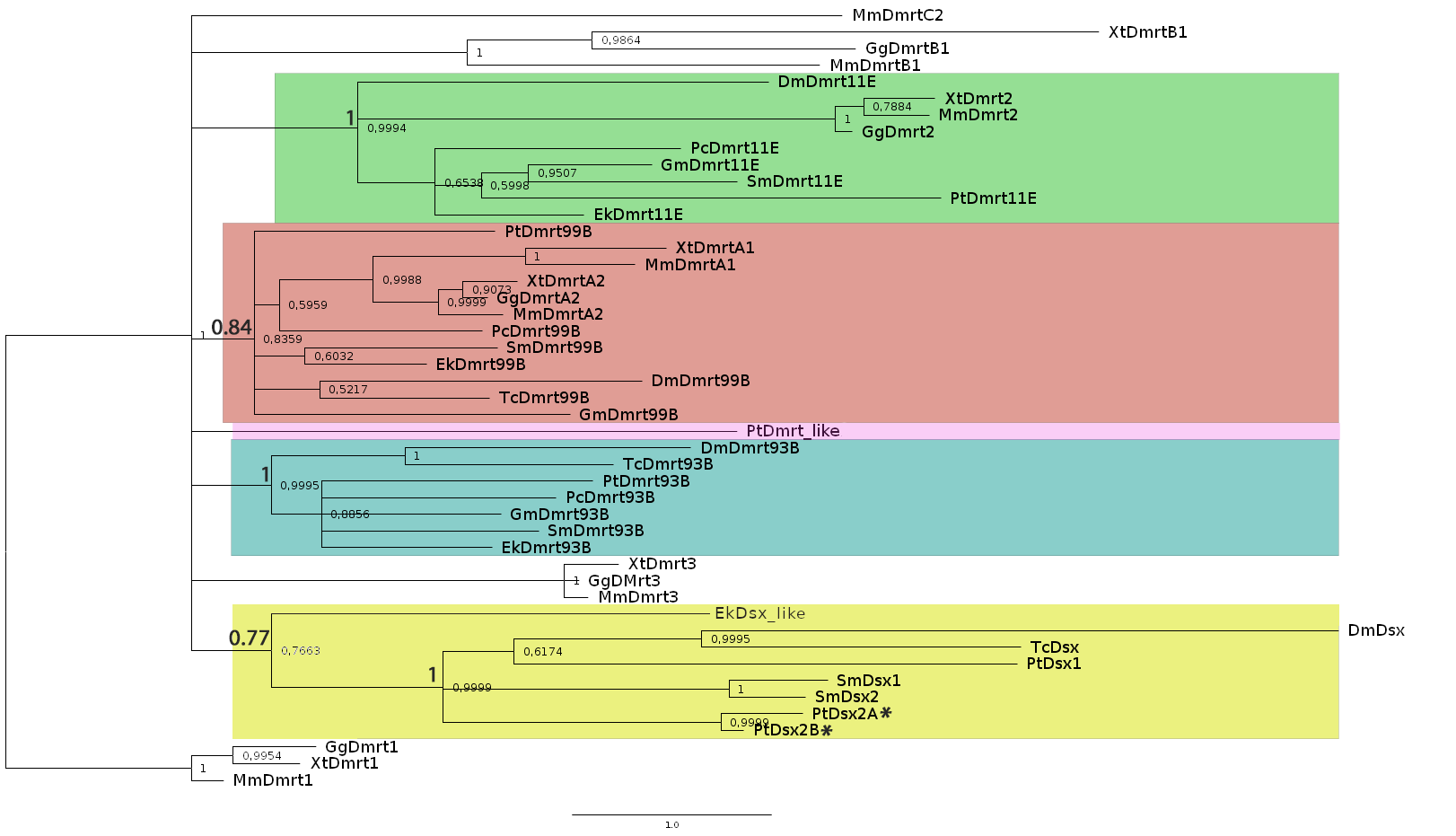

Supplement: Supplementary file 6 — Figure S3. Phylogenetic analysis based on the complete ORFs (with original branch length). (see Fig. 1 for further information). (TIF 4446 kb) [file 12983_2019_322_MOESM6_ESM.tif]

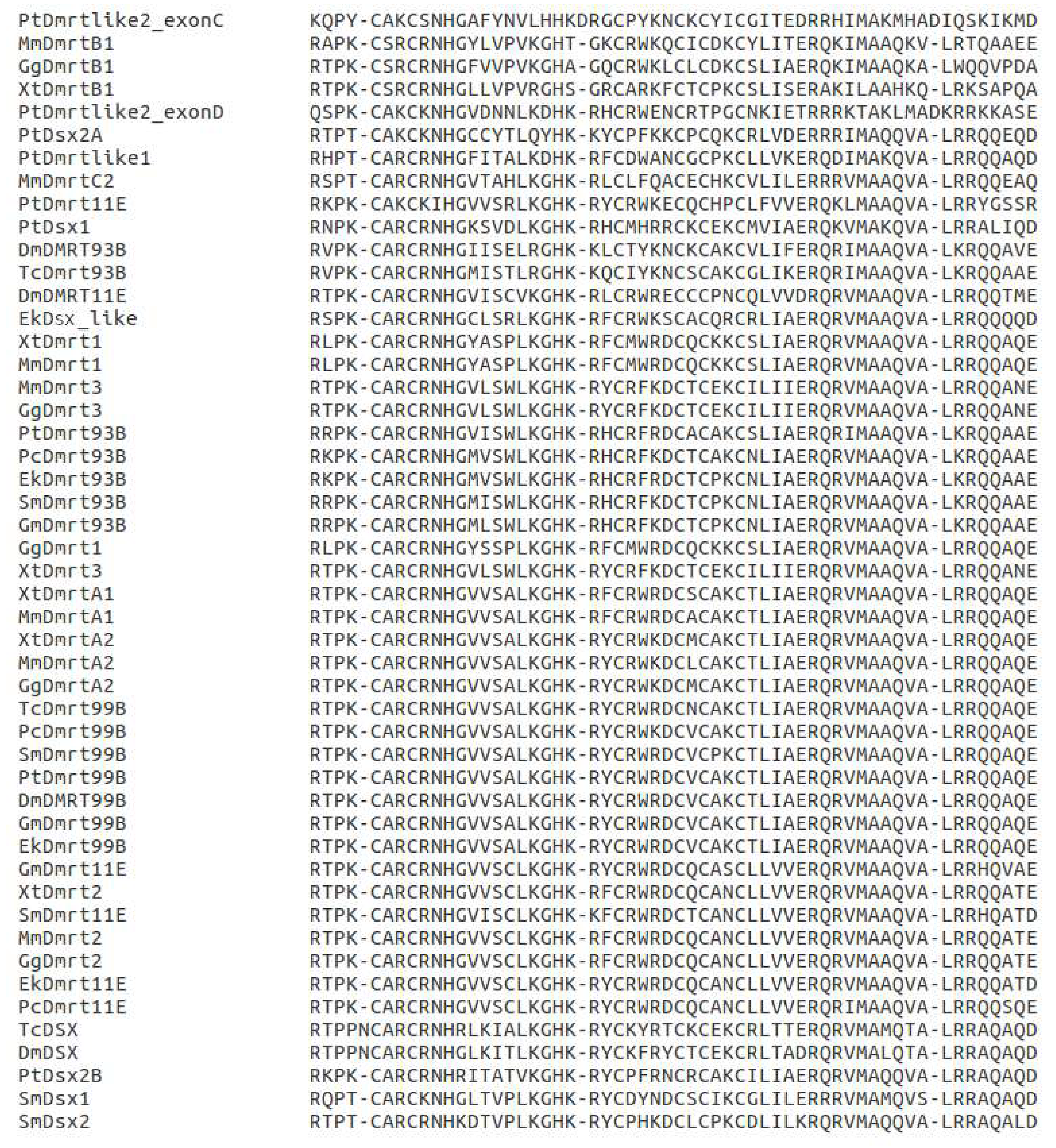

Supplement: Supplementary file 7 — Figure S4. Alignment of DM domains. (TIF 33987 kb) [file 12983_2019_322_MOESM7_ESM.tif]

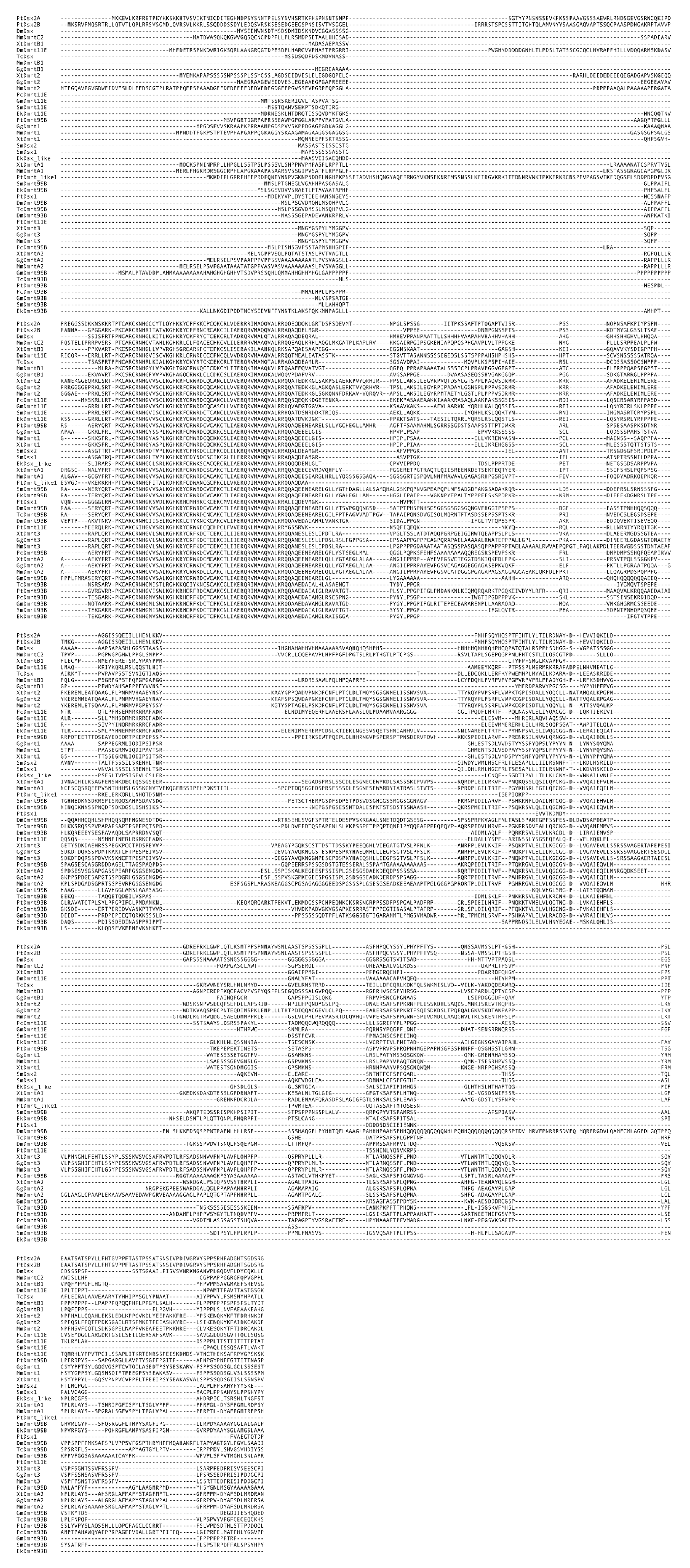

Supplement: Supplementary file 8 — Figure S5. Alignment of the complete ORFs. (TIF 35381 kb) [file 12983_2019_322_MOESM8_ESM.tif]

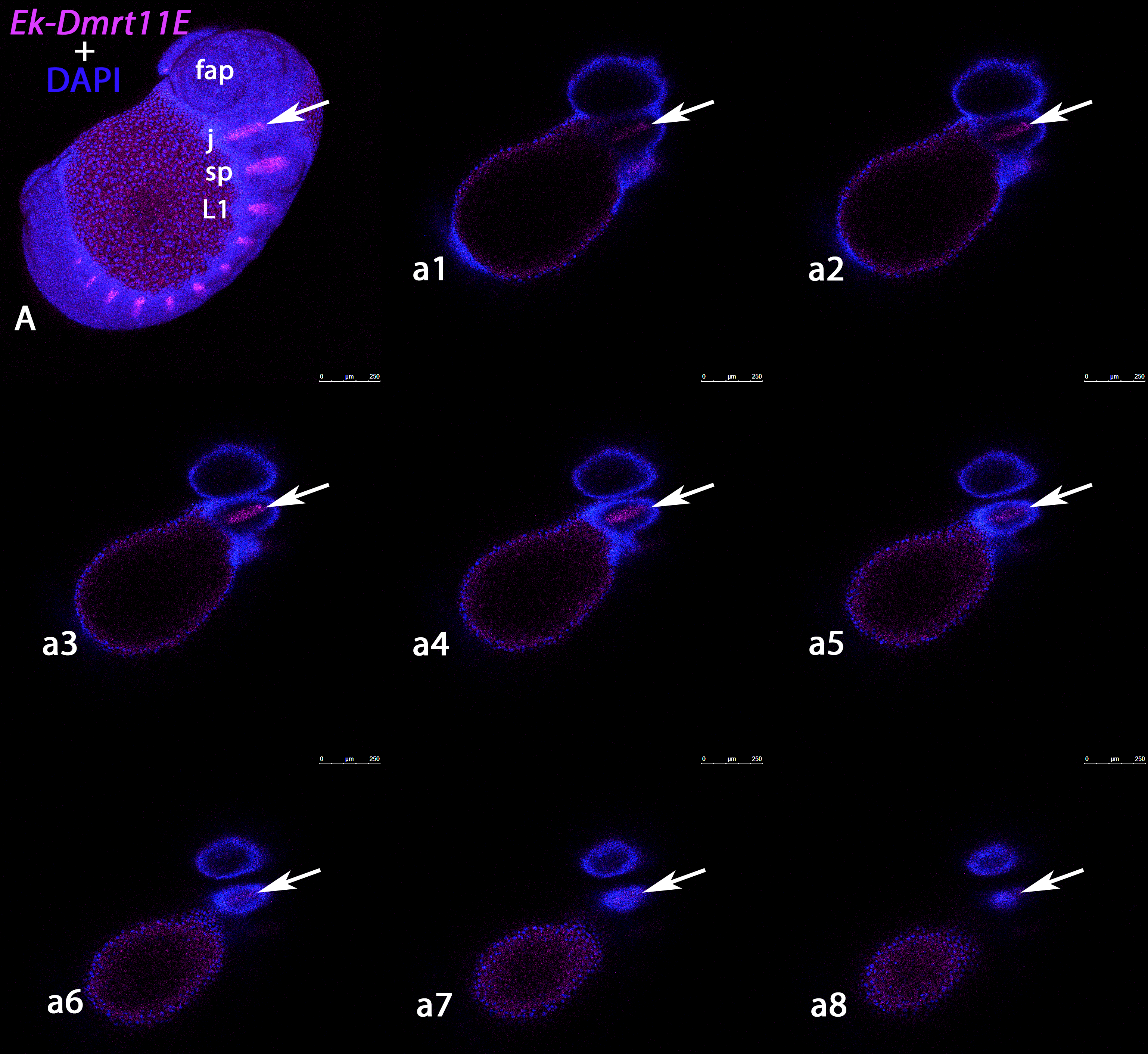

Supplement: Supplementary file 9 — Figure S6. Confocal data on the expression of Euperipatoides Dmrt11E. Magenta: Expression of Dmrt11E; Blue: DAPI. Panel A shows a Z-stack. Panels a1-a8 show a series of consecutive optical sections (6.5 μm per section) through part of the embryo. The focus is on the jaw-bearing segment (indicated by arrows). The data reveal that expression of Dmrt11E is exclusively inside the jaw, in mesodermal tissue, but not in the overlaying ectoderm. Abbreviations: fap, frontal appendage; j, jaw; L1, first walking-leg bearing segment; sp., slime papilla. (TIF 32533 kb) [file 12983_2019_322_MOESM9_ESM.tif]
